# Targeting ferroptosis to enhance the efficacy of mesenchymal stem cell-based treatments for intervertebral disc degeneration

**DOI:** 10.7150/ijbs.107021

**Published:** 2025-01-20

**Authors:** Yuzhu Xu, Xuanfei Xu, Renjie Chai, Xiaotao Wu

**Affiliations:** 1 Department of Spine Center, Zhongda Hospital, Medical School, Southeast University, Nanjing, Jiangsu, 210009, China.; 2 Department of Nuclear Medicine, Zhongda Hospital, Medical School, Southeast University, Nanjing, Jiangsu, 210009, China.; 3 Zhongda Hospital, School of Life Sciences and Technology, Advanced Institute for Life and Health, Southeast University, Nanjing, Jiangsu, 210009, China.

**Keywords:** Mesenchymal stem cell (MSC), MSC transplantation, Oxidative stress, Ferroptosis, Intervertebral disc degeneration (IVDD)

## Abstract

Although mesenchymal stromal cell (MSC) implantation shows promise for repairing intervertebral disc (IVD) degeneration (IVDD), their limited retention within degenerative IVDs compromises therapeutic efficacy. The oxidative stress in the microenvironment of degenerated IVDs induces a surge in reactive oxygen species production within MSCs, disrupting the balance between oxidation and antioxidation, and ultimately inducing ferroptosis. Recent evidence has suggested that targeting ferroptosis in MSCs could enhance MSC retention, extend the survival of transplanted MSCs, and markedly delay the pathological progression of IVDD. By targeting ferroptosis, a novel approach emerges to boost the efficacy of MSC transplantation therapy for IVDD. In this review, current research on targeting ferroptosis in MSCs is discussed from various perspectives, including the targeting of specific genes and pathways, drug preconditioning, and hydrogel encapsulation. A detailed discussion on the effects of targeting ferroptosis in MSCs on the transplantation repair of degenerated IVDs is provided. Insights that could guide improvements in stem cell transplantation therapies are also offered. Significantly, this review presents specific ideas for our future foundational research. These insights outline promising avenues for future clinical translation and will contribute to developing and optimizing treatment strategies for MSC transplantation therapy, maximizing benefits for patients with lumbar IVDD.

## 1. Introduction

The intervertebral disc (IVD) is a well-hydrated, fibro-cartilaginous tissue comprising a central, proteoglycan-rich nucleus pulposus (NP), an outer annulus fibrosus, and a cartilage endplate 1. It is critical for spinal mobility and stability 2. Located within the inner disc, NP cells (NPCs) are primarily responsible for synthesizing and maintaining the extracellular matrix (ECM) 3. The onset and acceleration of intervertebral disc degeneration (IVDD) are widely recognized to be initiated and accelerated by the loss of NPCs and the breakdown of the ECM 4. IVDD can lead to lower back pain which, if worsened, could result in disability and deteriorate the quality of life for patients 5. The condition involves mineralization and calcification, which reduce the IVD's nutrient supply and metabolism, thereby worsening IVDD 6-7. Although certain perspectives have suggested that directly targeting NPCs to boost their vitality could delay IVDD 8-9, transplanted NPCs often fail to proliferate sufficiently to produce adequate cell quantities or secrete sufficient functional ECM components owing to immune responses and limited self-renewal capability 2, 5, 10. Moreover, harvesting autologous NPCs could cause harm to the donor disc. Given these challenges, implanting NPC-based cells proves ineffective in regenerating degenerated NP tissue. Mesenchymal stem cell (MSC)-based therapy has emerged as a new field in regenerative medicine 11-12. MSCs, with their anti-inflammatory, regenerative, and immunomodulatory properties, are sourced from various tissues such as bone marrow, adipose tissue, the umbilical cord, and NP 1-2, 5. Transplanted MSCs migrate to damaged IVD tissue, differentiate into chondrogenic cell types, enhance proteoglycan production, and exhibit NPC characteristics 10, 13. Following differentiation into chondrogenic cell types, MSCs express high levels of chondrogenic markers such as type II collagen and aggrecan, key components of the ECM in the IVD 14-16. Thus, MSC transplantation could mitigate the progression of IVDD by supplementing depleted NPCs and exerting paracrine effects on compromised or aging NPCs 17-18. For instance, hypoxia-preconditioned MSCs maintain an NPC-like phenotype and curb IVD mineralization 19. Furthermore, the secretion of cytokines and growth factors by MSCs is crucial for enhancing the viability of resident NPCs, upregulating NP marker gene expression, and stimulating local tissue cells 14, 17.

However, the harsh and complex microenvironment of degenerative intervertebral discs (IVDs) poses a severe challenge to the survival of transplanted MSCs, thereby limiting the effectiveness of MSC-based therapies 14-15, 20-25. This adverse microenvironment is primarily due to excessive reactive oxygen species (ROS) and inflammation 21-22. The combination of excess ROS and inflammation leads to oxidative damage, reduces the retention rate of engrafted MSCs, and aimpedes their osteogenic differentiation, thus diminishing the efficacy of IVDD treatment. Additionally, the pathological progression in degenerated IVDs may impair the essential functions of MSCs. Elevated ROS levels contribute to protein carbonylation, lipid peroxidation, and DNA damage in engrafted MSCs 15. It is widely recognized that when pathological stimuli exceed their stress tolerance, MSCs undergo irreversible programmed cell death in such harsh environments 20, 23, 26-27. Consequently, enhancing the survival rate of MSCs under oxidative stress (OS) is essential for improving the efficacy of MSC-based therapy for IVDD. The severe oxidative stress in degenerated IVDs can induce various forms of programmed cell death in transplanted MSCs, including apoptosis, pyroptosis, and necrosis 27-32. These forms of regulated cell death are reportedly observed in MSCs under oxidative conditions 26-33. However, studies have shown that targeted strategies have not effectively mitigated the excessive loss of engrafted MSCs 26-27, 33. In MSC-based therapy for liver injury repair, poor retention of MSCs in the OS environment of the liver significantly diminishes their therapeutic efficacy 27. Notably, the addition of inhibitors of apoptosis, pyroptosis, and necrosis to MSCs under OS did not significantly delay the loss of stem cell retention. Similarly, our preliminary study on bone marrow mesenchymal stem cells (BMSCs) transplantation for repairing degenerated IVDs found that inhibiting apoptosis, pyroptosis, or necrosis did not significantly prevent BMSC death 26. Thus, it is plausible that other forms of regulated cell death induced by ROS stress significantly contribute to the excessive loss of MSCs in degenerated IVDs. In recent years, substantial evidence has established that OS-induced ferroptosis plays a crucial role in the survival and retention of transplanted MSCs, posing a significant threat 26-27, 34-38.

Iron, a critical trace element, is indispensable for maintaining redox reactions and homeostasis in organisms 39-40. Recent studies have shown that iron overload is an independent risk factor for human intervertebral disc degeneration (IVDD) and accelerates its pathological progression, indicating that transplanted MSCs are also exposed to this iron overload microenvironment 41-42. An iron overload microenvironment induces oxidative stress (OS) damage to MSCs and disrupts iron homeostasis within the stem cells, affecting the balance between oxidation and antioxidant systems. Ferroptosis, characterized by iron-dependent accumulation of lipid peroxides to lethal levels, threatens the survival of transplanted MSCs and results in poor MSC retention 34, 36-37. Our preliminary work has also confirmed that ferroptosis is responsible for the low MSC retention rate when engrafted into degenerative IVDs 26, 43. Therefore, targeting ferroptosis in MSC-based therapy is recognized as a promising approach for IVDD, and developing inhibitors of ferroptosis in MSCs may enhance the efficacy of MSC transplantation in degenerative IVDs.

## 2. MSC-based therapy for IVDD

First, MSC-based therapy has shown promise in preclinical treatments for IVDD. Yuan *et al.* 44 implanted cultured autologous BMSCs into degenerative IVDs and noted improved spinal segmental stability in the goat IVD defect model. Similarly, a rat IVDD model induced by needle puncture demonstrated that intradiscal injection of MSCs effectively restored the structural integrity and tissue composition of the IVD, highlighting the potential of MSCs in treating IVDD 20. Second, ongoing clinical trials on MSC-based therapy for IVDD have shown a moderate therapeutic effect 13, 45-53 (Table 1). Gomez-Ruiz *et al.* 13 reported on a 10-year follow-up of a prospective phase I**/**II clinical trial involving autologous MSC transplantation, noting improvements in radicular pain and low back pain (evaluated by the Visual Analog Scale (VAS) and the Oswestry Disability Index (ODI)). Lee *et al.* 53 evaluated the therapeutic effects of transplantation of adipose-derived stem cells (ADSCs) transplantation in patients with lumbar degenerative disc diseases, also reporting improvements in VAS pain scores, ODI scores, and radiological scores. Thus, these clinical trials support the efficacy of stem cell therapy for IVD regeneration. Current evidence suggests that stem cell transplantation therapy could be a viable option for partially restoring the biological characteristics of degenerated IVDs. However, ongoing concerns persist regarding the long-term safety and efficacy of MSC transplantation for repair treatments. Despite numerous benefits, potential long-term issues with MSC therapy include improper differentiation, immune suppression, and the risk of tumor formation 54-56. Cultured human MSCs may undergo spontaneous transformation under *in vitro* culture conditions, which do not accurately reflect the characteristics of MSCs in the bone marrow microenvironment *in vivo* 57. Consequently, their proliferation and differentiation capabilities may be affected, impacting the long-term efficacy of subsequent transplantation therapies. The primary concern remains that transplanted MSCs may undergo unwanted differentiation, potentially hindering anti-tumor immune responses and promoting new blood vessel formation, thereby facilitating tumor growth and metastasis 58-59. Despite significant progress in MSC therapy over the past few decades, many challenges remain. Therefore, large-scale follow-up studies are essential to confirm the long-term efficacy and safety of stem cell therapy, including the evaluation of stem cell immunocompatibility, stability, heterogeneity, differentiation, and migratory capacity.

Nevertheless, some studies have proposed exosomes secreted by MSCs as a safer alternative to MSC-based therapy 60-61. Stem cell-derived exosomes act as intercellular messengers and provide similar therapeutic effects to their parent MSCs, such as tissue regeneration, immunomodulation, and anti-inflammation 62-64. To date, only a few clinical studies have reported on the safety and potential efficacy of MSC-derived exosomes (MSC-Exos), including their use in preclinical and clinical studies of IVDD 65-66. Additionally, the rapid *in vivo* clearance of MSC-Exos and their relatively low extraction and purification efficiencies also limit their long-term therapeutic benefits 63. Given the dominance of MSC-based therapy in current clinical trials, this review will focus on stem cell transplantation therapy and summarize the relevant literature on targeting MSC ferroptosis to optimize preclinical strategies for the effective treatment of IVDD.

## 3. Targeting ferroptosis for MSC-based therapy

Ferroptosis is a form of regulated cell death (RCD) distinct from apoptosis 43, triggered by the accumulation of free intracellular iron and the disruption of redox regulatory mechanisms 67-68. It is characterized by chromatin condensation, disruption of membrane integrity, and morphologically evident by mitochondrial condensation, disappearance of mitochondrial cristae, and increased densities of the mitochondrial membrane 69-71.

**Figure 1:**
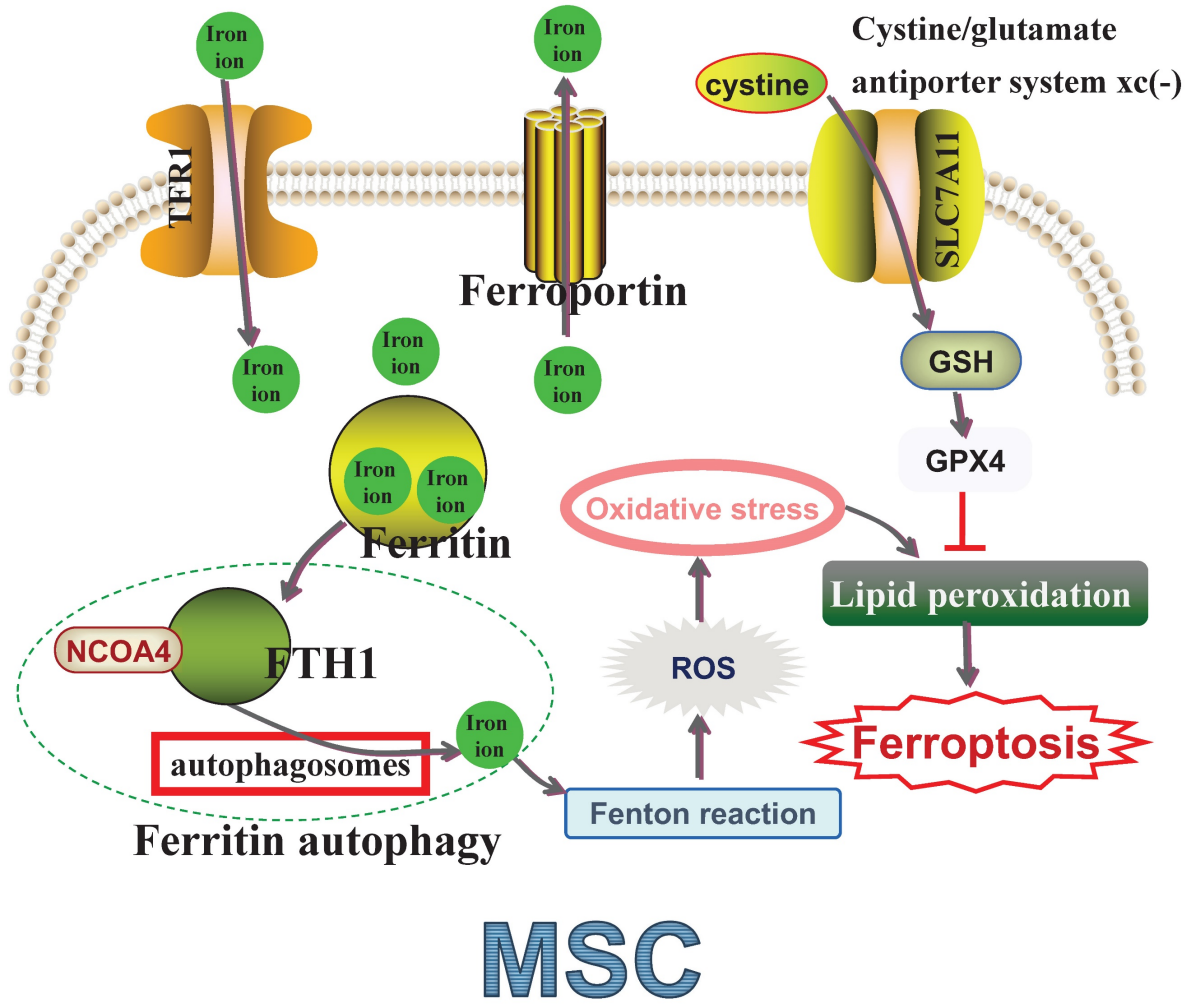
** Molecular mechanisms of ferroptosis in MSCs.** TFR1: Transferrin receptor protein 1; NCOA4: nuclear receptor coactivator 4; FTH1: ferritin heavy chain 1; ROS: Reactive oxygen species; SLC7A11: Solute carrier family 7 member 11; GSH: Glutathione synthetase; GPX4: Glutathione peroxidase 4; MSC: Mesenchymal stem cell.

Transferrin receptor protein 1 (TFR1) is responsible for iron uptake, while ferroportin manages iron export, both essential for maintaining intracellular iron homeostasis 72-73. Ferritin, comprising ferritin heavy chain 1 (FTH1) and ferritin light chain (FTL), also helps maintain iron homeostasis by storing iron to prevent toxicity 73-74. Nuclear receptor coactivator 4 (NCOA4) mediates 'ferritin autophagy', where NCOA4 binds FTH1 and facilitates ferritin delivery to autophagosomes for lysosomal degradation, releasing sequestered iron 75. This process leads to the generation of reactive oxygen species (ROS) via the iron-based Fenton reaction, and excessive ROS production, often referred to as oxidative stress (OS), accelerates lipid membrane oxidation and causes lipid bilayer membrane rupture, ultimately inducing ferroptosis 76-77. Hence, lipid peroxidation is considered a crucial indicator of OS 78-79. Solute carrier family 7 member 11 (SLC7A11) is vital in the cystine/glutamate antiporter system xc(-), which transports cystine intracellularly for glutathione synthetase (GSH) biosynthesis 80. Glutathione peroxidase 4 (GPX4) uses antioxidant GSH as a cofactor to detoxify lipid peroxidation effectively and prevent ferroptosis 81-82 (Figure 1). Increasing evidence suggests that targeting ferroptosis could significantly enhance MSC-based therapies 26-27, 35-36, 83-85 (Table 2). A deeper understanding of ferroptosis mechanisms in MSCs is crucial for developing strategies to improve their functionality and survival, ultimately optimizing cell-based therapy outcomes.

### 3.1 Inhibiting MSC ferroptosis via the iron metabolism pathways

Ferroptosis is implicated in the OS-induced loss of transplanted MSCs under harsh degenerative conditions in intervertebral discs (IVDs), with this involvement closely regulated by intracellular iron metabolism 34-36. Iron ions disrupt the redox balance when accumulated excessively within cells and tissues, catalyzing ROS generation and promoting ferroptosis 39-40. In response to the initiation mechanisms of ferroptosis, researchers have developed strategies encompassing iron uptake, storage, and transport to preserve cellular iron homeostasis and prevent MSC ferroptosis. In an OS injury *in vitro* model, Jing *et al.* 37 demonstrated significant activation of the ferroptosis pathway through transcriptomic analysis and phenotypic experiments. Notably, while NCOA4 expression levels increased and ferritin levels decreased, their binding rate was found to be elevated, suggesting that NCOA4-mediated ferritin degradation contributes to iron accumulation and promotes ferroptosis.

In a rat model of smoking-related osteoporosis, inhibitors of ferritinophagy or ferroptosis were shown to suppress BMSC ferroptosis, enhance cell viability, and thereby reduce BMSC dysfunction and reverse bone damage 37. Similarly, in dental pulp stem cells, NCOA4-mediated ferritinophagy has been linked to ferroptosis, indicated by cytosolic iron overload, elevated ROS levels, and increased NCOA4 expression 86. Knocking down NCOA4 was observed to reduce ferritin degradation. Additionally, another study targeting the iron storage pathway showed potential in inhibiting MSC ferroptosis and enhancing MSC survival rates. In an *in vitro* model of BMSC ferroptosis induced by erastin, overexpression of FTH1/FTL was found to reduce ROS levels and abrogate lipid peroxidation, markedly improving BMSC survival 87. This finding indicates that targeting the FTH1**/**FTL pathway could enhance resistance to ferroptosis and improve engrafted BMSC survival. Moreover, other studies have employed pre-treatment of stem cells with deferoxamine (DFO). DFO, known as a ferroptosis inhibitor, chelates cytoplasmic iron ions, preventing their interaction with peroxides and thus inhibiting the formation of harmful radicals 88. Hopfner *et al.* 89 found that DFO-preconditioned ADSCs significantly enhanced regenerative capabilities for wound healing in diabetes while reducing intracellular ROS levels, offering a new approach to improve diabetic ADSC functionality. Khoshlahni *et al.* 90 also reported that DFO-preconditioned BMSCs resisted OS damage in stressful environments. Through iron depletion by DFO, BMSCs showed improved survival rates under OS conditions, facilitating subsequent BMSC-based transplantation therapies. These findings underscore the importance of modulating the iron metabolism pathway in maintaining iron homeostasis and inhibiting MSC ferroptosis. We were also acutely aware of the critical role of targeting MSC ferroptosis in clinical MSC treatments.

Therefore, we hypothesized that regulating iron metabolism in MSCs influences the efficiency of stem cell transplantation in repairing degenerated IVDs. Free ferrous iron (Fe **^2+^**) acts as a potent oxidative factor, generating unstable radicals via the Fenton reaction 76, 91. Coenzyme Q10 (Co-Q10) has been reported to reduce the perferryl radical, chelate iron, and thus prevent iron overload 92-93. Lazourgui *et al.* 92 confirmed that Co-Q10 helps regulate iron levels, improving cellular antioxidant status and alleviating oxidative stress (OS) during insulin resistance. Peng *et al.* 94 observed that intracellular iron ion levels, when overloaded, corresponded with a continuous decrease in Co-Q10, exacerbating cellular OS, further suggesting Co-Q10's efficacy as an iron chelating agent. In a rat needle puncture IVDD model, Sun *et al.* 15 administered Co-Q10 preconditioned BMSCs into degenerative IVDs, noting improved viability, restored mitochondrial structure and function, and increased production of ECM components. Thus, Co-Q10 protects against OS-induced BMSC ferroptosis, providing a protective role in cellular survival and differentiation, enhancing the efficacy of BMSC transplantation therapy, and may represent a viable treatment option for IVDD. Similarly, adding ferritin, crucial for iron homeostasis and detoxification, has shown cytoprotective effects on BMSCs and promoted their differentiation into osteoblasts, essential for bone tissue health 95-96. The protective mechanism likely involves enhancing the iron ion storage pathway, effectively countering OS-induced BMSC ferroptosis. However, direct *in vivo* research evidence is currently lacking regarding the regulation of intracellular iron levels in stem cells and its impact on the efficiency of repairing degenerated IVDs. More importantly, future studies in both cellular and rat IVDD models will aim to demonstrate that interventions in the iron metabolism pathway can desensitize transplanted MSCs to ferroptosis, further validating the regulation of ferroptosis by imbalanced iron homeostasis. Targeting iron-chelating drugs, iron metabolism, or redox-related proteins may offer a new strategy to enhance the efficiency of MSC-based therapy in IVDD.

### 3.2 Inhibiting MSC ferroptosis via the antioxidant pathways

It is widely acknowledged that oxidative stress (OS) is a primary pathogenic factor in the numerous pathophysiological processes of IVDD 8, 97-102. Studies have shown that OS accelerates IVDD, and the OS microenvironment within degenerated discs jeopardizes the survival of transplanted MSCs 23, 97, 99. OS is characterized by an imbalance between ROS production and scavenging 8, 98. Iron overload, overwhelming the antioxidant defense system, initiates the classic ferroptosis pathway, closely linking ferroptosis to OS 81, 103-107. Park *et al.* 108 reported that excessive ROS production activates OS-induced lipid peroxidation, thereby exacerbating ferroptosis-dependent cytotoxicity in human astrocytes. Sánchez-Ortega *et al.* 107 observed that excessive ROS production also induces ferroptosis in lung squamous cell carcinoma (LUSC), suggesting that targeting OS-induced LUSC ferroptosis could present a new therapeutic approach. Increasing evidence indicates that molecular pathways activated by OS intersect with those of ferroptosis in transplanted MSCs, sharing several molecular targets such as accumulation of lipid peroxidation products, and low levels of GSH and GPX4 103, 105-106. Persistent and excessive OS in the transplantation site triggers ferroptosis in MSCs due to elevated ROS levels, significantly reducing MSC retention after transplantation. Accordingly, targeting ferroptosis could markedly improve the effectiveness of stem cell transplantation therapy by reducing intracellular OS levels or enhancing antioxidant defenses. For example, in an erastin-treated BMSC model, the antioxidant quercetin demonstrated effective anti-ferroptosis properties through its antioxidative functions, protecting BMSCs from erastin-induced ferroptosis 109.

Tert-butyl hydroperoxide (TBHP) has been shown to induce cellular oxidative injury, and we used TBHP to simulate OS conditions in an *in vitro* model 26, 110-111. TBHP treatment significantly increased ferroptosis indicators (Fe^2+^ aggregation, increased ROS levels, excessive lipid peroxidation) and altered levels of ferroptosis-related proteins (Long-chain-fatty-acid--CoA ligase 4 (ACSL4), Prostaglandin G/H synthase 2 (PTGS2), GPX4, and Ferritin) 26. Conversely, ROS scavengers and ferroptosis inhibitors (ferrostatin-1 (Fer-1) and liproxstatin-1 (Lip-1), both potent lipid peroxidation inhibitors) suppressed the increase in OS-induced ferroptosis indicators and blocked the expression of ferroptosis-related proteins 26. Therefore, previous studies and our preliminary findings collectively confirm that OS can activate ferroptosis, and suggest that employing antioxidants or activating antioxidant pathways could mitigate OS-induced MSC ferroptosis.

#### 3.2.1 Activating KEAP1/Nrf2/GPX4 signaling pathway

As a primary defender against ferroptosis, upregulation of GPX4 levels can render MSCs resistant to ferroptosis. Hu *et al.* 27 identified ferroptosis as the cause of poor MSC retention rates shortly after exposure to an OS microenvironment or following engraftment into an injured liver setting. Strategies to suppress MSC ferroptosis include pretreating MSCs with Fer-1 and Lip-1 and enhancing intracellular GPX4 transcription 27. These approaches have effectively increased MSC retention under ROS stress and improved the liver-protective outcomes post-implantation. Conversely, BMSCs influenced by neuroblastoma displayed increased sensitivity to ferroptosis, due to GPX4 downregulation 112. Similarly, pretreatment of BMSCs with poliumoside, which boosts intracellular GPX4 expression levels, effectively countered OS-induced BMSC ferroptosis, as indicated by reduced mitochondrial ROS and malondialdehyde (MDA) levels, and increased GPX4 protein levels 84. In contrast, disrupting GPX4 expression through lentiviral transfection diminished the anti-ferroptosis effects.

The nuclear factor erythroid 2-related factor 2 (Nrf2), a transcription factor located in the mammalian nucleus, regulates the stress-induced activation of cytoprotective genes, including GPX4 106, 113-114. Bhat *et al.* 115 mechanistically demonstrated that enhancing Nrf2 transcriptional activity could prevent lipid peroxidation and reduce suppression of GPX4 expression during ferroptosis. Furthermore, activating the Nrf2-dependent GPX4 antioxidant pathway in doxorubicin-stimulated H9c2 cardiomyocytes markedly alleviated ferroptosis and mitochondrial damage 113. In addition, Nrf2 nuclear translocation is negatively regulated by Kelch-like ECH-associated protein 1 (KEAP1), and the KEAP1**/**Nrf2 complex can modulate intracellular OS levels 107, 116. In cardiomyocytes, reducing KEAP1 levels promoted the nuclear translocation of Nrf2 and transcription of SLC7A11 and GPX4, thus offering protection against doxorubicin-induced ferroptosis 113. Koppula *et al.* 117 also confirmed that KEAP1transcriptionally regulates Nrf2 levels, and the KEAP1/Nrf2 pathway regulates ferroptosis via a GPX4-independent mechanism. In an erastin-induced BMSC ferroptosis *in vitro* model, the import of excess iron via TFR1 and minimal export by ferroportin, alongside a dysregulated KEAP1**/**Nrf2**/**GPX4 axis and low SLC7A11 levels, collectively exacerbated BMSC ferroptosis 118. Deactivating the KEAP1**/**Nrf2**/**GPX4 pathway reduced BMSC ferroptosis, as indicated by decreased KEAP1 protein levels and increased Nrf2 and GPX4 protein levels. The cystathionine γ-lyase (CSE)**/**hydrogen sulfide (H**_2_**S) pathway is vital for redox homeostasis, including ROS scavenging, antioxidant activation, and cellular protection against OS 119-120. The CSE**/**H**_2_**S pathway is crucial in modulating ferroptosis 121-122. Regulation of this pathway in MSCs has been shown to protect against ferroptosis, improving low retention and engraftment rates post-MSC delivery. Notably, enhancing the CSE**/**H**_2_**S pathway induced Keap1 S-sulfhydration, activating Nrf2 and inhibiting ferroptosis, as evidenced by reduced iron levels and ROS production, and increased GPX4 protein levels 122. Thus, the CSE**/**H**_2_**S pathway exerts anti-ferroptosis effects by mediating the KEAP1**/**Nrf2**/**GPX4 in MSCs, ultimately enhancing MSC survival post-delivery into mice 122.

Targeting ferroptosis has proven effective in enhancing the therapeutic efficacy of endogenous MSCs for treating IVDD. Increasing GPX4 expression in NP-derived MSCs (NPSCs) inhibited NPSC ferroptosis and promoted their proliferation, revealing significant therapeutic potential for endogenous MSCs in IVDD treatment 123. Additionally, activating Nrf2 in ADSCs effectively alleviated stem cell dysfunction and the cell death rate in degenerative IVDs and stimulated ADSC differentiation into an NPC-like phenotype 124. This suggests that targeting ferroptosis could enhance ADSC transplantation therapy for IVDD and is promising for improving stem cell transplantation efficacy. Although direct evidence is lacking for modulating the KEAP1/Nrf2/GPX4 pathway to inhibit MSC ferroptosis in treating IVDD, existing evidence supports enhancing the KEAP1**/**Nrf2**/**GPX4 axis in MSCs for use in preclinical and clinical trials of MSC-based therapies for IVDD.

#### 3.2.2 Preconditioning of MSCs with antioxidants

In addition to targeting antioxidant pathways within MSCs, recent studies have utilized direct pre-treatment with antioxidants to protect MSCs from ferroptosis. Quercetin was shown to inhibit erastin-induced BMSC ferroptosis through antioxidant pathways, potentially converting into its metabolite quercetin diels-alder anti-dimer (QDAD) during this process 109. Both quercetin and QDAD exhibit strong antioxidant and anti-ferroptosis properties. Ebselen, an antioxidant with glutathione peroxidase-like activity, was found to prevent BMSC ferroptosis and reduce its inhibitory effect on osteogenic differentiation of BMSCs 125. Curcumin, known for its potent antioxidant properties, enhanced the antioxidant capacity of MSCs, as indicated by increased survival rates 83. Curcumin preconditioning also reduced MSC death in the hostile brain microenvironment and improved MSC therapeutic efficacy for treating intracerebral hemorrhage. Curcumin-preconditioned MSCs exhibited neuroprotective effects in an intracerebral hemorrhage model, characterized by reduced cellular injury and lower ROS levels in neuronal cells 83. Geraniin showed ferroptosis-inhibitory effects in BMSCs induced by erastin, involving inhibition of lipid peroxidation, iron chelation, and antioxidant actions 126. Picein, possessing antioxidant properties, ameliorated erastin-induced oxidative stress and enhanced the proliferation and migration of BMSCs 85. The protective mechanism of Picein involves activating the Nrf2**/**Heme oxygenase 1 (HO-1)**/**GPX4 pathway. HO-1, regulated by Nrf2 and associated with antioxidant proteins, helps counter oxidative stress by facilitating Nrf2 nuclear translocation to bind the antioxidant response element of the HMOX1 gene 127-131. Activation of the Nrf2**/**HO-1 pathway increases GPX4 levels to counter OS 132-133, highlighting the importance of the Nrf2/HO-1/GPX4 axis in countering BMSC ferroptosis. This class of antioxidants similarly exerts ferroptosis-inhibiting effects by activating intracellular antioxidant pathways. However, Yuan *et al.* 35 presented a contrasting view, noting an upregulation of HO-1 in macrophages that increased intracellular Fe 2+ and promoted ferroptosis, indicating that the anti-ferroptosis effects of Picein in BMSCs require further exploration.

Growing evidence has shown that antioxidant preconditioning effectively enhances MSC proliferation, migration, and yields better therapeutic outcomes for repairing degenerated intervertebral discs (IVDs). Icariin, a flavonoid with anti-ferroptotic activity, mitigates oxidative stress damage and promotes BMSC osteogenesis 134. Icariin treatment has been shown to improve the therapeutic efficacy of stem cell transplantation for repairing degenerated IVDs by alleviating pathological trends of IVDD and increasing collagen II and aggrecan levels in IVD tissues 134. Neochlorogenic acids, possessing antioxidant and anti-ferroptosis properties, potentially act through the Nrf2**/**HO-1 pathway 135. These acids have been shown to reduce hydrogen peroxide (H**_2_**O**_2_**)-induced ROS production in BMSCs, thereby inhibiting ferroptosis. *In vivo*, BMSCs preconditioned with neochlorogenic acids exhibited enhanced protective effects for IVDD compared to other model groups, attributed to improved BMSC stability during the repair process 135. Similarly, co-encapsulating BMSCs with salvianolic acid B, a potent antioxidant, in 1% hyaluronic acid methacrylate hydrogel significantly reduced cell death rates compared to the BMSCs **+** hydrogel group 136. It was observed that salvianolic acid B efficiently reduced ferroptotic damage caused by H**_2_**O**_2_** to BMSCs and increased cell survival percentages *in vitro*. In a rat model, pretreatment of BMSCs with salvianolic acid B delayed disc degeneration progression compared to stem cell therapy alone, as evidenced by histological and immunohistochemical analyses 136. Thus, encapsulating BMSCs with salvianolic acid B presents potential research value for regenerative disc tissue repair. In summary, these findings collectively support the use of MSC transplantation with antioxidants as a viable approach for advancing MSC-based tissue engineering for IVDD repair, potentially through ferroptosis inhibition. This also offers a new perspective on suppressing MSC ferroptosis to increase the retention of grafted MSCs and enhance the efficiency of stem cell transplantation repair.

### 3.3 Inhibiting MSC ferroptosis by targeting specific molecular and pathway

#### 3.3.1 HIF-1α

Hypoxia-inducible factor 1-alpha (HIF-1α) was identified as a key ferroptosis gene by analyzing differentially expressed genes from OS-induced BMSCs compared to controls, based on the Gene Expression Omnibus databases and a ferroptosis dataset 137-138. The pivotal role of HIF-1α in regulating ferroptosis was further validated in an animal model, suggesting that targeting HIF-1α to suppress MSC ferroptosis might be a novel approach for treating IVDD 137. Previous studies have shown that a hypoxic microenvironment stabilizes cellular HIF-1α and enhances ferroptosis resistance in a HIF-1α-dependent manner, likely through increased HIF-1α expression and its interaction with hypoxia-inducible factor 1 beta to form the HIF-1 complex 139-140. Activation of the HIF-1**/**hypoxic response elements (HRE) signaling pathway then promotes the binding of HRE to the solute carrier family 2, facilitated glucose transporter member 12 (SLC2A12) promoter, elevating SLC2A12 expression, modulating glutathione metabolism, and conferring resistance to ferroptosis 140. Transplanting ADSCs into degenerative IVDs demonstrates the therapeutic potential of MSC-based therapy for IVDD, although the physiological hypoxic state and OS contribute to low retention of transplanted ADSCs 141-142. Hypoxia-preconditioned ADSCs have shown increased cell proliferation and migration capabilities and promoted differentiation into NPC-like cells via HIF-1α, whereas inhibiting HIF-1α produced the opposite effect 143. He *et al.* 144 observed significant decreases in HIF-1α levels and NPSC counts in degenerative rat and human IVDs. Similarly, hypoxia-preconditioned NPSCs demonstrated enhanced resistance to cell death by activating HIF-1α, involving HMOX1 and solute carrier family 2, facilitated glucose transporter member 1 144. Overexpressing HIF-1α in NPSCs also resulted in higher survival rates post-transplantation into degenerative discs *in vivo* 144. These findings collectively support the anti-ferroptosis effect of HIF-1α under the severe hypoxic and OS conditions typical of degenerative discs, suggesting HIF-1α's crucial role in enhancing the therapeutic efficacy of MSCs for IVDD treatment. However, other research presents a contrary view, indicating that inhibition of HIF-1α enhanced the anti-ferroptosis effects of Fer-1 in chondrocytes 145, and downregulation of HIF-1α reduced ferroptosis and improved gastric and minor intestinal mucosal injury in an animal model 138, 146. Therefore, the approach of targeting HIF-1α to suppress MSC ferroptosis remains controversial, and extensive foundational research on MSC-based therapy for IVDD is essential to elucidate this strategy.

#### 3.3.2 SIRT1

NAD-dependent histone deacetylase sirtuin-1 (SIRT1) is a nicotinamide adenine dinucleotide-dependent enzyme that regulates critical metabolic proteins during oxidative stress (OS) 147-148. The SIRT1**/**Nrf2 signaling pathway provides anti-OS effects and can prevent ferroptosis, restoring redox balance. Sanz-Alcázar *et al.* 149 reported that frataxin deficiency in dorsal root ganglion neurons disrupts iron homeostasis, reduces SIRT1 expression, diminishes Nrf2 activation, and impairs the cellular response to OS, ultimately leading to ferroptosis. Conversely, increased SIRT1 levels activate the liver kinase B1 homolog**/**5'-AMP-activated protein kinase pathway, which in turn activates Nrf2, playing a vital role in the antioxidant response 149. Activation of the Sirt1/Nrf2/GPX4 pathway also protects BMSCs from erastin-induced ferroptosis and enhances cell viability, thereby improving the effectiveness of BMSC-based therapy 36. Interestingly, in fundamental research on MSC-based therapy for IVDD, activating SIRT1 reduced ROS accumulation, decreased expression of senescence-related proteins, and increased the proliferation ability of NPSCs 150. SIRT1 activation also confirmed the efficacy of NPSCs in alleviating IVDD in a puncture-induced IVDD rat model based on *in vivo* assessments 150. However, inhibiting SIRT1 partially reversed the therapeutic effects of NPSCs on degenerated IVDs 150-151. Further research showed that overexpressing SIRT1 in MSCs effectively mitigated IVDD, as evidenced by the recovery of IVD height and volume and increased mRNA and protein levels of type II collagen and aggrecan, which inhibit the NF-kappaB p65 inflammatory pathway 152. Additionally, activating the SIRT1**/**PPAR-γ co-activator 1α pathway, closely associated with mitochondrial function and antioxidant activities 147, 153, alleviated OS-induced mitochondrial dysfunctions, including reduced mitochondrial ROS production, increased mitochondrial membrane potentials, and enhanced survival rates of transplanted NPSCs, thus enhancing the therapeutic potential of MSCs for IVDD 154. Overexpression of SIRT1 could mitigate some limitations in MSC survival and adaptation under the severe conditions of disc degeneration, potentially due to SIRT1-mediated anti-ferroptosis effects in MSCs. Collectively, these findings indicate that targeting SIRT1 holds promise for treating IVDD by improving the therapeutic efficacy of MSC-based therapy.

#### 3.3.3 PI3K/AKT pathway

ROS accumulation in degenerative IVDs can trigger ferroptosis in MSCs and reduce the survival rate of engrafted MSCs. Suppressing MSC ferroptosis could potentially enhance their survival in the oxidative stress (OS) microenvironment of degenerative IVDs. Further mechanistic studies have shown that the phosphatidylinositol 3-kinase (PI3K)**/**threonine-protein kinase (AKT) signaling pathway can modulate ferroptosis, offering a novel approach for stem cell therapy in treating IVDD 155-156. The PI3K**/**AKT signaling cascades are activated by hormones, growth factors, and other extracellular stimuli to regulate essential cellular functions such as cell proliferation, regulated cell death (RCD), and cell survival 157-159. For example, activation of the PI3K**/**AKT pathway provides neuroprotection by phosphorylating Nrf2, which then enhances FTH1 transcription to store excess iron ions and prevent iron toxicity from excessive Fe^2+^ accumulation 160. Furthermore, activation of the PI3K/AKT pathway has been shown to increase optic atrophy 1 expression, a member of the mitochondrial fusion protein family, promoting mitochondrial fusion and preventing mitochondrial structural and functional abnormalities, ultimately reducing ferroptosis 155, 161-162. The PI3K**/**AKT pathway, associated with resistance mechanisms and cell survival, can be activated to desensitize cells to ferroptosis, offering an alternative approach to stem cell therapy for IVDD. In an OS microenvironment *in vitro* model of BMSCs, the PI3K**/**AKT signaling pathway was found to be involved in OS-induced BMSC ferroptosis 163. The upregulation of PI3K and AKT phosphorylation increased anti-oxidative gene expression and reduced intracellular ROS levels, thereby inhibiting BMSC ferroptosis and enhancing cell viability 163. Interestingly, activation of the PI3K**/**AKT signaling pathway also improved the antioxidant defense of BMSCs under the OS microenvironment and enhanced their osteogenic differentiation capacity, as demonstrated by increased mRNA transcripts of osteogenic markers 164. Activating the PI3K**/**AKT pathway has improved anti-OS processes in MSCs by suppressing ferroptosis, providing a theoretical foundation for enhancing stem cell-based therapy for treating IVDD by increasing the survival of transplanted MSCs in the OS microenvironment.

NPSCs can differentiate into NPCs and exert paracrine effects that maintain the quantity and quality of IVD cells, thus enhancing stem cell-based therapy for intervertebral disc regeneration 144, 165. Activation of the PI3K**/**AKT pathway promotes NPSC proliferation and adaptation to the niche of degenerated IVDs, advancing the endogenous repair process 166-167. In an *in vitro* oxidative stress (OS) microenvironment simulated by H2O2, activation of the PI3K**/**AKT pathway alleviated mitochondrial dysfunction, including changes in mitochondrial ultrastructure and mitochondrial ROS production, thereby reducing ROS levels and increasing NPSC viability 166. Conversely, pretreatment of NPSCs with a PI3K inhibitor diminished these protective effects and exacerbated NPSC ferroptosis. In rat models of mechanical loading stress-induced IVDD, excessive mechanical loading inactivated the PI3K**/**AKT pathway in human NPSCs, resulting in intracellular ROS accumulation, mitochondrial dysfunction, and decreased cell viability 168. This validated the role of the PI3K**/**AKT signaling pathway in regulating and inhibiting ferroptosis. Activation of the PI3K/AKT pathway reversed these effects and efficiently suppressed ferroptosis in NPSCs. Significantly, activation of the PI3K/AKT pathway alleviated cell death in human NPSCs *in vivo* models and substantially mitigated IVDD 168. The structure of the IVD was restored, the amount of extracellular matrix (ECM) increased, and cell numbers were augmented. Furthermore, in a rat IVDD model induced by needle puncture, activation of the PI3K/AKT pathway reduced ROS generation and maintained mitochondrial homeostasis in NPSCs, while inhibitors of this pathway attenuated these protective effects 165. The PI3K**/**AKT pathway alleviated excessive cell death of NPSCs induced by OS in the microenvironment of degenerative IVDs, as evidenced by X-ray, magnetic resonance imaging (MRI), and histological analyses 165. Thus, the PI3K**/**AKT signaling pathway emerges as a promising candidate for treating IVDD by increasing the survival rate of NPSCs. Therefore, suppressing MSC ferroptosis via the PI3K/AKT pathway could be adopted for IVDD treatment, establishing a specific therapeutic strategy to preserve MSC viability and potentially enhance IVD regeneration.

#### 3.3.4 GDFs

Growth differentiation factors (GDFs) modulate cellular processes such as proliferation, differentiation, and cell death 169-170. Previous research has indicated that GDFs may mediate oxidative stress (OS) responses and be involved in ferroptosis 171-172. For example, in a mouse model of sepsis-induced cardiomyopathy, GDF-15 provided protection to cardiomyocytes by inhibiting OS and suppressing ferroptosis, thereby reducing myocardial injury 173. Further research showed that GDF-15 promotes the transcription of GPX4, reducing lipid peroxidation in cardiomyocytes. In another model, GDF-15 knockout in ferroptosis induced by erastin led to decreased intracellular glutathione (GSH) levels and accelerated ferroptosis progression 174. This study also demonstrated that GDF-15 enhances the expression of SLC7A11, explaining its role in increasing intracellular GSH and GPX4 levels. In a sepsis model induced by cecal ligation and puncture in mice, overexpression of GDF-11 inhibited ferroptosis by upregulating SIRT1, thereby reducing lung tissue damage and inflammation, and preserving alveolar barrier integrity, thus presenting a promising molecular target for acute lung injury treatment 175. These findings underscore the role of GDFs in maintaining redox balance and regulating ferroptosis in BMSCs.

GDF-5, part of the transforming growth factor-beta superfamily, enhances chondrogenic differentiation 176-177. In an oxidative stress environment simulating IVDD, adding GDF-5 reduced OS-induced cell death in NPSCs and promoted their chondroid differentiation. This effect is possibly mediated by the RhoA**/**Rho-associated protein kinase (ROCK) signaling pathway 176. In models of myocardial injury and subarachnoid hemorrhage in mice and rats, modulation of the RhoA**/**ROCK pathway has been shown to influence cardiomyocyte and neuronal ferroptosis, respectively 178-179. This suggests that GDF-5 may enhance MSC survival by suppressing ferroptosis through the RhoA/ROCK pathway, indicating potential for gene-targeted NPSCs. Additionally, in a rat tail IVDD model, GDF-5-preconditioned BMSCs significantly improved NP regeneration and outperformed the sole BMSC treatment 180. Okoro *et al.* also reported that GDF-5 enhanced differentiation of BMSCs into NP-like cells, supporting the NPC phenotype 181. A series of experiments suggests that GDF-5 not only inhibits ferroptosis in engrafted MSCs but also effectively induces their differentiation into NP-like cells both *in vivo* and *in vitro*, offering a feasible approach for NP regeneration and IVDD repair. In summary, targeting MSC ferroptosis using GDF-5 as a gene target has shown great potential and warrants further validation in additional *in vivo* IVDD animal models.

#### 3.3.5 Activating Prominin-2 exerts anti-ferroptosis effects

As a 100-kDa glycoprotein, Prominin-2 comprises five transmembrane domains and two glycosylated extracellular loops 182-183. It is a member of cholesterol-binding proteins and is associated with plasma membrane protrusions 184. PROM2 (encoding the prominin 2) mRNA is expressed in various normal human tissues 185. Recently, Prominin-2 has been recognized as a ferroptosis inhibitor that can transport ferritin out of the cell, thereby reducing intracellular iron ion accumulation and preventing ferroptosis 182, 186-188. Adamiec-Organisciok *et al.* 187 confirmed that PROM2 is a ferro-resistance marker, as evidenced by increased PROM2 mRNA levels in resistant cell lines, and decreased levels in sensitive cell lines. Paris *et al.* 186 discovered that overexpression of Prominin-2 is associated with ferroptosis resistance by reducing cytoplasmic Fe **^2+^** accumulation, thus reducing lipid peroxidation and ferroptosis. Brown *et al.* 189 reported that Prominin-2 opposes ferroptotic cell death. They found that 4-hydroxynonenal, a lipid peroxidation by-product and a known ferroptosis biomarker, activates heat shock factor protein 1 (HSF1), which induces Prominin-2 expression through HSF1-dependent transcription of PROM2. Additionally, activating transcription factor 1 enhances ferroptosis resistance by upregulating N6-adenosine-methyltransferase subunit transcription, thereby stabilizing PROM2 mRNA 184. Increased Prominin-2 reduces erastin-induced ferroptosis and improves cell viability and proliferation rate 184. These findings demonstrate that Prominin-2-activated iron export contributes to ferroptosis resistance and has significant implications for enhancing MSC-based therapy for IVDD.

Based on this research, we proposed that targeting the mechanism inducing Prominin-2 expression could expand the options to enhance MSC ferroptosis resistance. Activating Prominin-2 expression could be a viable approach for improving the therapeutic efficiency of MSC-based therapies for IVDD. Our preliminary work supports that ferroptosis is a major factor in the early, rapid, and extensive depletion of BMSCs under *in vitro* ROS stress, consistent with the aforementioned findings 26. Additionally, we transfected BMSCs with an overexpression lentivirus to generate Prominin-2-overexpressed BMSCs. We also confirmed that activating Prominin-2 expression significantly mitigates the loss of BMSCs during the initial stage after implantation into degenerative IVDs. Enhanced BMSC retention slows the depletion of engrafted BMSCs and effectively boosts their therapeutic efficacy in the harsh environment of degenerative IVDs. Although our previous findings are impactful for MSC-based therapy, targeted Prominin-2 activators are not yet available.

**Figure 2:**
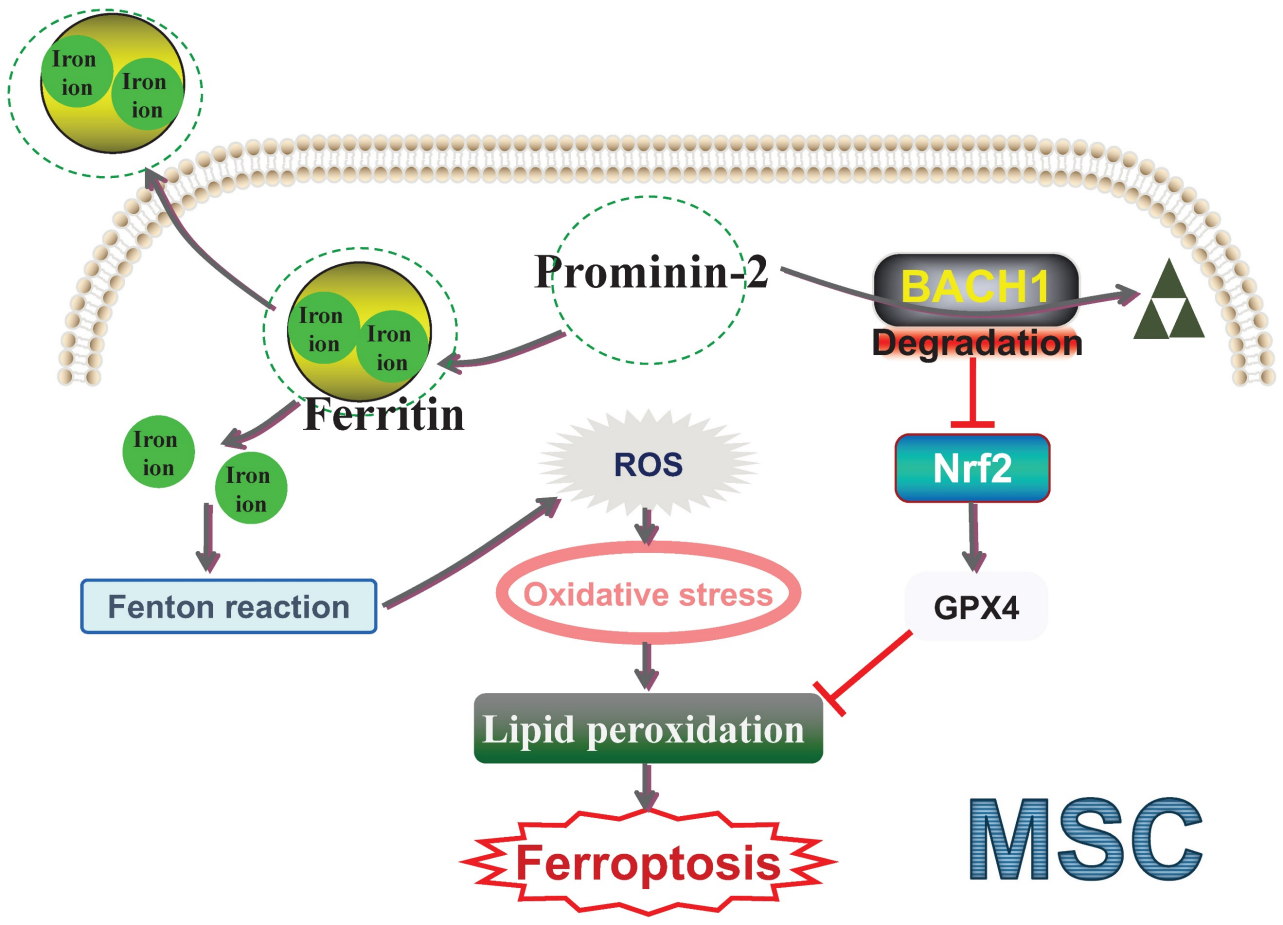
** Prominin-2 exhibits dual anti-ferroptosis effects effects MSC ferroptosis.** ROS: Reactive oxygen species; BACH1: BTB and CNC homolog 1; Nrf2: Nuclear factor erythroid 2-related factor 2; GPX4: Glutathione peroxidase 4; MSC: Mesenchymal stem cell.

In MSC-based therapy for IVDD, targeting Prominin-2-mediated ferroptosis defense offers a potential intervention. Our future work will delve into the involvement and underlying mechanisms of Prominin-2 in ferroptosis and reveal its additional roles. For instance, our preliminary work discovered that Prominin-2 not only transports iron ions beyond the cell but also physically interacts with BTB and CNC homolog 1 (BACH1) and facilitates its degradation 26. BACH1, a transcription factor, represses multiple antioxidant genes (including Nrf2) and thus disrupts cellular redox homeostasis 190-192. Increasing evidence indicates that BACH1 enhances ferroptosis by inhibiting the transcription of various OS-induced protective genes 193-195. Regarding its mechanism, Prominin-2 exerts a dual role in OS-induced ferroptosis through the iron metabolism pathway and the Prominin-2**/**BACH1 antioxidant pathway (Figure 2). Investigating the upstream and downstream nodes of this pathway could clarify the function of Prominin-2 and provide new insights for Prominin-2-activating drug discovery. Moreover, evidence has shown that hydrogels could boost MSC survival after engraftment in the adverse OS microenvironment 196-197. In this context, we also plan to explore the potential of Prominin-2 activator-delivering hydrogels to enhance MSC resistance to OS. Activating Prominin-2 could prevent ferroptotic cell death of transplanted MSCs and improve the therapeutic effect of MSCs for IVDD. In summary, a deeper exploration of the anti-ferroptosis mechanism based on Prominin-2 is highly promising.

### 3.4 Hydrogel-encapsulated stem cells exert anti-ferroptosis effects

Given the harsh OS microenvironment in degenerative IVDs sensitizes MSCs to ferroptosis, targeting transplanted MSC ferroptosis is essential for enhancing the clinical efficacy of cell transplantation therapy for IVDD. This includes modulating the ferroptosis signaling pathway in stem cells or enhancing the intracellular antioxidant defense system. It is important to note that injecting gene-modified or antioxidant preconditioned MSCs might cause a sudden increase in intradiscal pressure, contributing to the leakage of engrafted MSCs. Considering the challenge of injecting MSCs into degenerative IVDs, an optimal candidate carrier could be designed to address this challenge. Composite hydrogels exhibit good biocompatibility, are injectable for minimally invasive administration, and maintain stable mechanical strength after injection to prevent MSC leakage 198-200. More importantly, hydrogel encapsulation creates a defensive shield for the transplanted MSCs and protects them from ferroptotic damage, thus reducing ferroptosis in stem cells 33, 35, 201. *In vivo* research on aged bone regeneration showed that the fabricated injectable hydrogel was highly sensitive to ROS and effectively scavenged intracellular ROS of BMSCs in the OS microenvironment 199. This demonstrated that composite hydrogels efficiently modulate the antioxidant function of BMSCs to defend against ferroptosis and improve the host microenvironment, thereby enhancing BMSCs' self-renewal ability and osteogenic capacity. Additionally, composite hydrogels could also be utilized for sustained drug release, including ferroptosis inhibitors and antioxidants 35, 202-203. The sustained drug release from the hydrogel has a persistent and strong anti-ferroptosis effect on MSCs. In a rat-infected bone defect model, ferroptosis was identified as the primary cause of BMSC death under the infected bone microenvironment. Based on this, Yuan *et al.* 35 designed a hydrogel composite scaffold with ROS-responsive and anti-ferroptosis properties, featuring long-term Fer-1 release to deliver BMSCs for repairing infected bone defects. Targeting ferroptosis mitigated OS damage to BMSCs and protected their cell viability, thus preserving osteogenic differentiation potential and facilitating osteogenic regeneration. Results from micro-CT, X-ray, and histological evaluations indicated that the hydrogel composite scaffold-loaded BMSCs targeted ferroptosis to better promote bone regeneration than the pure hydrogel group and are a promising therapeutic approach for repairing infected bone defects. As a result, composite hydrogels for MSC encapsulation provide a direct protective barrier and continuously inhibit ferroptosis through the OS pathway within the stem cells. Alternatively, it can achieve sustained and controlled release of ferroptosis inhibitors, thus achieving continuous suppression of ferroptosis.

Increasing numbers of *in vivo* studies on IVDD show that composite hydrogels carrying MSCs are promising for enhancing the effectiveness of stem cell-based therapy 14, 20, 66, 204. These hydrogels have shown anti-ferroptosis effects on engrafted MSCs, thus promoting IVD regeneration. In rat models of IVDD, ADSCs or ADSC-laden hydrogels were transplanted into degenerative IVDs 124. Four weeks post-implantation, the retention of ADSCs in the composite hydrogels was higher, and the proportion of the NP area was also larger than in other groups. Notably, there was a significant increase in cell proliferation rate and a decrease in intracellular MDA levels in the ADSC-laden hydrogel group, indicating that hydrogel-encapsulated ADSCs effectively resisted ferroptotic damage under OS conditions. More importantly, these hydrogels showed a more substantial effect in delaying IVDD, preserving IVD tissue integrity, and boosting NP-like ECM production 124. In another study using a rat IVDD model, MSCs were encapsulated by alginate and gelatin microgel, and the biocompatible hydrogel loaded with MSCs was delivered into degenerative IVDs 20. It is noteworthy that damage to mitochondrial cristae was reduced under transmission electron microscopy in the hydrogel-encapsulated group, demonstrating that ferroptosis contributed to engrafted MSC death under OS conditions. Hydrogel encapsulation protected MSCs in the harsh disc microenvironment, prolonged MSC retention, and preserved their migration, proliferation, and differentiation properties, ultimately resulting in more effective reduction of disc degeneration compared to treatment with MSCs alone 20. Based on this defense against MSC ferroptosis after transplantation, Wang *et al.* designed a manganese oxide (MnOx) nanohydrogel to deliver BMSCs to repair IVDD 125. MnOx has strong antioxidant enzyme activities and can continuously eliminate ROS in degenerative IVDs and improve the OS microenvironment 205-206. This injectable composite nanohydrogel enables MnOx to be more resistant to degradation, allowing it to maintain effective concentrations in the lesion area for extended periods. Importantly, MnOx nanohydrogel reduced ferroptotic damage to BMSCs, as evidenced by increased cell proliferation, enhanced BMSC metabolic activity, and lowered intracellular ROS levels 207. Due to its superior anti-ferroptosis effects, the BMSCs released by hydrogels maintain high stemness and low senescence under OS conditions, and also remodel NPCs' ECM. *In vivo*, injection of BMSC-loaded MnOx nanohydrogel showed the highest therapeutic efficacy, as assessed by gross evaluation, X-ray and MRI examinations, and histological staining 207. In conclusion, the biocompatible MSC hydrogel suppressed ferroptosis activation and improved MSC survival after intradiscal transplantation. Targeting the ferroptosis signaling pathway, combined with hydrogel encapsulation, shows great potential and could be an effective strategy to enhance the effectiveness of MSC-based therapy for treating IVDD diseases.

## 4. Research prospects

Existing evidence suggests that cell therapy is a feasible method for partially restoring the biological characteristics of degenerated IVDs. MSCs, noted for being non-tumorigenic and easily accessible, are commonly used as cell therapy source cells. Reports indicate that intradiscal engraftment of MSCs can boost the accumulation of proteoglycans and collagen, leading to improved radiological results and pain alleviation. However, the effectiveness of unmodified MSCs is largely limited. The ineffective therapeutic outcomes of MSC therapy are attributed to the OS microenvironment within the degenerated IVDs, which is characterized by ischemic and hypoxic stress that disrupts the balance between the synthesis and degradation of the ECM. It is increasingly clear that simply implanting stem cells *in vivo* is insufficient to maximize their healing capabilities. Therefore, protecting the engrafted MSCs and maintaining their physiological functions is critical to enhancing the efficacy of MSC-based therapy for IVDD. Moreover, inhibiting transplanted MSC cell death to promote MSC retention is essential. The harsh OS microenvironment could trigger apoptosis, necrosis, and pyroptosis in MSCs 27-32. However, studies have shown that inhibiting apoptosis, necrosis, and pyroptosis could only partially restore the vitality of transplanted MSCs 26-27, 33. It seems that other RCD pathways might also contribute to transplanted MSC death. The process leading to ferroptosis activation involves a complex disruption of cellular iron balance, resulting in elevated cytosolic metal ion levels that cause OS and irreversible damage to cell structures and biomolecules. Notably, the harsh OS microenvironment within degenerated IVDs compromises the balance between oxidative and antioxidative systems in MSCs, making them prone to ferroptosis. Therefore, ferroptosis-associated cell death could further reduce the therapeutic effects of MSC therapy. Conversely, protecting MSCs from ferroptosis could enhance the efficacy of MSC treatment. Increasing evidence suggests that targeting ferroptosis for stem cell transplantation therapy effectively enhances their retention within degenerated IVDs. By manipulating stem cells to intervene in ferroptosis, researchers and clinicians could further boost MSC survival rate and provide a significant advantage during the initial stages of *in vivo* placement, where MSCs face numerous challenges. Hence, suppressing transplanted MSC ferroptosis and enhancing their preservation under the harsh microenvironment of the discs are crucial for optimizing MSC-based transplantation therapy. This review focused on current ferroptosis-related strategies for enhancing *in vivo* cell survival and subsequent tissue regeneration by manipulating stem cells or their surrounding environment.

**Figure 3:**
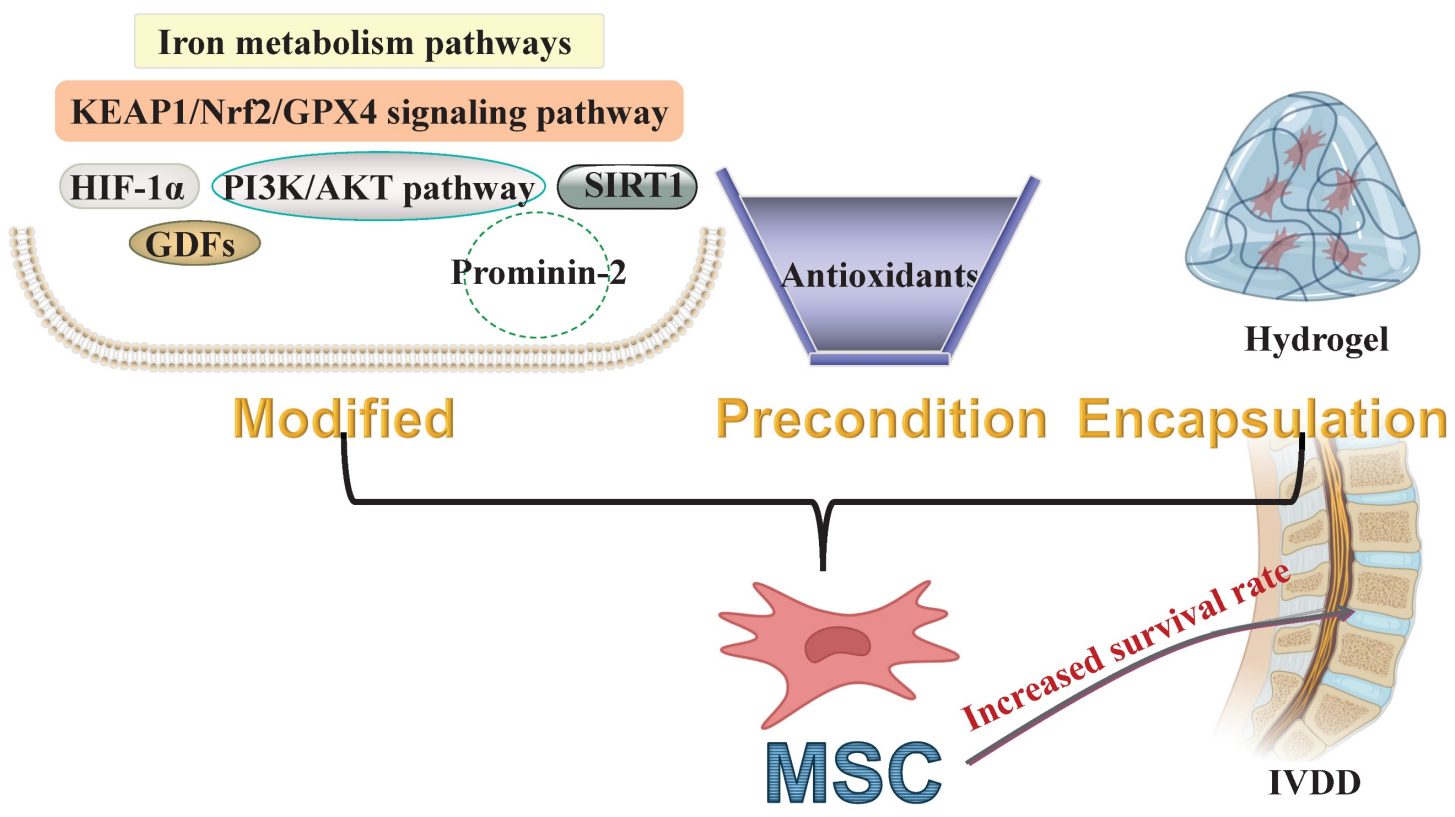
** Developing preconditioning strategies for MSCs to increase the survival rate of transplanted MSCs within the degenerated IVDs.** KEAP1: Kelch-like ECH-associated protein 1; Nrf2: Nuclear factor erythroid 2-related factor 2; GPX4: Glutathione peroxidase 4; HIF-1α: Hypoxia-inducible factor 1-alpha; PI3K: Phosphatidylinositol 3-kinase; AKT: Threonine-protein kinase; SIRT1: NAD-dependent histone deacetylase sirtuin-1; GDFs: Growth differentiation factors; MSC: Mesenchymal stem cell; IVDD: Intervertebral disc degeneration.

The MSCs to the host microenvironment is critical for successful stem cell-based transplantation therapies. Strategies for preconditioning, including genetic modification, drug preconditioning, and hydrogel encapsulation, have been developed to enhance the adaptation and functionality of MSCs in pathological contexts. Recent advances in targeted ferroptosis treatments have proved beneficial in both *in vitro* and *in vivo* models of IVDD, employing stem cell transplantation. Moreover, these treatments are associated with reduced ROS accumulation and the activation of antioxidant pathways. Preconditioning MSCs through iron metabolic pathways can effectively inhibit ferroptosis by reducing intracellular ROS levels. The application of antioxidants during preconditioning significantly enhances the adaptability of engrafted MSCs to the harsh conditions of the IVD. This improvement may be due to the activation of the Nrf2/GPX4 signaling pathway. Non-canonical molecules, such as HIF-1α, SIRT1, PI3K**/**AKT, GDFs, and Prominin-2, modulate the ferroptosis process by influencing the balance between oxidation and antioxidation within MSCs. Therefore, reducing intracellular iron content and enhancing antioxidant defenses are key mechanisms for improving the adaptability of preconditioned MSCs to stressful environments. Developing MSC preconditioning strategies based on these aspects could enhance the survival rate of transplanted MSCs within the degenerated IVDs, better utilizing their intrinsic properties for repair (Figure 3). In our review of preliminary clinical studies on stem cell transplantation for the repair of degenerated IVDs, various tissue-derived stem cells were considered. MSCs can be obtained from various tissues, such as bone marrow, umbilical cord, adipose tissue, each exhibiting distinct phenotypic and functional features 208. Although these stem cells from different sources have shown some repair effectiveness, comparing the results of various studies is challenging. One possible reason for this issue is the inherent and extensive heterogeneity among these stem cells 54. This variation in tissue sources may also contribute to inconsistencies in the outcomes of future clinical applications. As MSC-based therapies progress through multiple clinical trials, it is crucial to implement strategies to minimize product heterogeneity. Therefore, a standardized evaluation of the specific functions of MSCs is necessary in the future (for example, defining the optimal tissue source), moving towards more consistent and effective MSC-based therapy for IVDD 209. Moreover, there is currently a lack of studies that reproducibly and reliably confirm the potential of targeted MSC ferroptosis in early simulated clinical settings. Although targeted ferroptosis strategies have improved the regenerative effects of these stem cells from different sources in transplantation repair of degenerated IVDs, their preclinical and early clinical efficacy may be inconsistent and often requires verification in later trials. The susceptibility of these stem cells from different sources to ferroptosis varies in the OS environment of degenerated IVDs, leading to different retention rates after transplantation. Therefore, evaluating the efficacy of targeted MSC ferroptosis more effectively and reliably is essential. Approaches to address these differences may include carefully selecting tissue sources, donors, and specific MSC subpopulations, as well as standardized cultivation conditions and potency evaluations 209-210. This is also a key focus area of our future research.

This review outlines the potential applications of targeting MSC ferroptosis through iron metabolism and antioxidant pathways, critical molecules involved in ferroptosis, and MSC encapsulation with hydrogels. The delivery of preconditioned or genetically modified MSCs, combined with composite hydrogels, creates a three-dimensional protective environment that more effectively inhibits ferroptosis. It is particularly important to develop strategies that fully leverage the anti-ferroptosis effects of Prominin-2 in MSCs through dual-pathway inactivation of ferroptosis. Based on our previous research, activating Prominin-2 maintains intracellular iron ion homeostasis and may also repress BACH1 expression to regulate antioxidant pathway in MSCs. However, excessive and prolonged OS prevents Prominin-2 from effectively suppressing BACH1 expression in MSCs due to the cellular response to transcription factor BACH1 190-191. The BACH1 inhibitor hemin can effectively degrade BACH1, offering potential for regulating antioxidant metabolism within stem cells 211-212. We envision combining these three strategies to target MSCs effectively, enhancing targeted ferroptosis effects. We plan to design a hydrogel composite scaffold that provides a cellular protective barrier and allows for sustained delivery of the BACH1 inhibitor hemin. This is expected to enable gene-modified MSCs to acquire barrier protection directly in the transplantation OS microenvironment and continuously inhibit intracellular BACH1 expression, thereby more effectively suppressing ferroptosis in MSCs through both oxidative and antioxidative pathways. A growing body of evidence confirms that MSCs preconditioned by targeting ferroptosis show potential for IVD regeneration in preclinical models, where engrafted MSCs increased disc height, upregulated ECM production, and elevated the expression of NP marker genes. In summary, targeted ferroptosis preconditioning is promising for promoting MSC adaptation to OS microenvironments, and introducing ferroptosis-targeting drugs can help MSCs survive in these environments and enhance their repair efficiency for IVDD.

## 5. Conclusion

Supported by preclinical and clinical studies, MSC-based cell therapy has emerged as a promising option for IVDD diseases and is gradually entering clinical practice. However, the low retention of MSCs in the harsh microenvironment is a major reason for their loss and suboptimal therapeutic outcomes after transplantation into degenerative IVDs. The OS microenvironment triggers a surge in intracellular ROS, destabilizing the balance between oxidation and antioxidation. This imbalance is a key pathway for ferroptosis and has been linked to the loss of MSC retention. Clinical translation strategies that target ferroptosis in MSCs could improve retention, prolong survival, and boost therapeutic outcomes. Our review emphasized the potential benefits of targeting ferroptosis inhibition in MSCs for treating IVDD diseases, drawing on a comprehensive assessment of basic research and early clinical translation. We also provided a comprehensive overview of pertinent basic research and early clinical translational studies. We believe that targeting ferroptosis in MSCs could offer new perspectives for the future treatment of IVDD diseases. However, there is currently a lack of clinical translational research focusing on targeting ferroptosis in MSCs to enhance the efficiency of degenerated IVD repair. In future research, we need to further confirm *in vivo* the impact of targeting ferroptosis on MSC retention rate and the efficiency of repairing degenerated IVDs. Additionally, there is an urgent need for specific biomarkers that are common to both MSCs and ferroptosis to accurately predict the efficiency of MSC ferroptosis inhibition. Moreover, the data from primary research still needs to meet the standards required for clinical application. Therefore, more effective strategies for inhibiting MSC ferroptosis need to be developed. Nevertheless, targeting MSC ferroptosis holds promise for novel therapies for IVDD diseases.

## Figures and Tables

**Table 1:** Clinical studies of MSC therapy for IVDD

Location	Model	Cell type	Indication	N	Outcome
Spain 45 2019 Feb	Autologous	BMSCs	Lumbar degenerative disc diseases	11	5-year follow-up; Improvement of low back pain and on radiograph
Sweden 46 2019 Sep	Autologous	MSCs	Lumbar degenerative disc diseases	4	8-month or 28-month follow-up; MSCs have differentiated into chondrocyte-like cells
Spain 47 2021 Feb	Allogenic	BMSCs	Lumbar degenerative disc diseases	12	3.5-year follow-up; Improvement of low back pain and on MRI
American 48 2021 Feb	Allogenic	Mesenchymal precursor cells	Lumbar degenerative disc diseases	60	36-month follow-up; Improvement of low back pain
American 49 2022 Mar	Autologous	BMSCs	Lumbar degenerative disc diseases	36	12-month follow-up; Improvement of low back pain and leg pain
China 50 2022 Oct	Autologous	ADSCs	Lumbar degenerative disc diseases	75	24-month follow-up; The research is ongoing
Japan 51 2023 Feb	Allogenic	BMSCs	Lumbar spinal canal stenosis	30	96-week follow-up; The research is ongoing
American 52 2023 Mar	Allogenic	Umbilical cord stem cells	Lumbar degenerative disc diseases	33	26.88-month follow-up; Improvement of low back pain and leg pain
Spain 13 2023 Apr	Autologous	BMSCs	Lumbar degenerative disc diseases	11	10-year follow-up; Improvement of low back pain and on radiograph
Republic of Korea 53 2023 Nov	Autologous	ADSCs	Lumbar degenerative disc diseases	8	6-month follow-up; Improvement of low back pain and on MRI

**Table 2:** Targeting ferroptosis for MSC-based therapy

Authors Year	Cell type	Intervention strategy	Detection	Animal model	Results of *in vivo* experiments
Hu *et al.* 27 2023 May	ADSCs	Pretreat MSCs with ferroptosis inhibitors and upregulate intracellular GPX4 transcription	Increase MSC survival rate	The rats' injured liver milieu	Improve MSC therapeutic efficacy in the injured liver
Huang *et al.* 83 2023 Jul	OM-MSCs	Pretreat MSCs with antioxidant	Enhance the anti-oxidative activity of MSCs	The rats' intracerebral Hemorrhage	Show better neuroprotective effects
Xu *et al.* 26 2023 Nov	BMSCs	Pretreat BMSCs with ferroptosis inhibitors and overexpress Prominin-2	Increase BMSC survival rate	The rats' IVD degeneration	Improve MSC therapeutic efficacy in the degenerative IVD
Xu *et al.* 84 2024 Mar	BMSCs	Pretreat BMSCs with ferroptosis inhibitor and antioxidant	Increase BMSC survival rate	The murine type 2 diabetic osteoporosis model	Revitalize bone density, curtail ROS abundance, and amplify GPX4 presence in the distal femur
Huang *et al.* 36 2024 May	BMSCs	Developed bone graft substitute material to load MSC	Increase BMSC survival rate	The osteoporotic bone defect rat model	Alleviate the inflammatory environment and promote bone regeneration in osteoporotic bone defect
Huang *et al.* 85 2024 July	BMSCs	Pretreat BMSCs with antioxidant	Increase BMSC survival rate	The osteoporosis rat model	Reduce the inflammation levels and promote bone regeneration
Yuan *et al.* 35 2024 Aug	BMSCs	Design an anti-ferroptosis 3D-printed hydrogel scaffold loaded with BMSCs	Increase BMSC survival rate	The rat model of infected bone defects	Capable of eradicating pathogens and promoting bone regeneration

Olfactory mucosa-derived mesenchymal stem cells (OM-MSCs)

## Abbreviations

ADSCs: Adipose-derived stem cells
BMSCs: Bone marrow mesenchymal stem cells
BACH1: BTB and CNC homolog 1
Co-Q10: Coenzyme Q10
CSE: Cystathionine γ-lyase
DFO: Deferoxamine
ECM: Extracellular matrix
FTH1: Ferritin heavy chain 1
Fer-1: Ferrostatin-1
Fe ^2+^: Ferrous iron
FTH1: Ferritin heavy chain 1
FTL: Ferritin light chain
GPX4: Glutathione peroxidase 4
GSH: Glutathione synthetase
GDFs: Growth differentiation factors
HSF1: Heat shock factor protein 1
HO-1: Heme oxygenase 1
H_2_O_2_: Hydrogen peroxide
H_2_S: Hydrogen sulfide
HIF-1α: Hypoxia-inducible factor 1-alpha
HRE: Hypoxic response elements
IVD: Intervertebral disc
IVDD: Intervertebral disc degeneration
KEAP1: Kelch-like ECH-associated protein 1
Lip-1: Liproxstatin-1
ACSL4: Long-chain-fatty-acid--CoA ligase 4
LUSC: Lung squamous cell carcinoma
MRI: Magnetic resonance imaging
MnOx: Manganese oxide
MSC: Mesenchymal stem cell
MSC-Exos: Mesenchymal stem cell-derived exosomes
SIRT1: NAD-dependent histone deacetylase sirtuin-1
Nrf2: Nuclear factor erythroid 2-related factor 2
NCOA4: Nuclear receptor coactivator 4
NP: Nucleus pulposus
NPCs: Nucleus pulposus cells
NPSCs: Nucleus pulposus-derived mesenchymal stem cells
ODI: Oswestry Disability Index
OS: Oxidative stress
PI3K: Phosphatidylinositol 3-kinase
PROM2: encoding the prominin 2
PTGS2: Prostaglandin G/H synthase 2
QDAD: Quercetin diels-alder anti-dimer
ROS: Reactive oxygen species
RCD: Regulated cell death
ROCK: Rho-associated protein kinase
SLC2A12: Solute carrier family 2, facilitated glucose transporter member 12
SLC7A11: Solute carrier family 7 member 11
TBHP: Tert-butyl hydroperoxide
AKT: Threonine-protein kinase
TFR1: Transferrin receptor protein 1
VAS: Visual Analog Scale

## References

[1] Swahn H, Mertens J, Olmer M, Myers K, Mondala TS, Natarajan P (2024). Shared and Compartment-Specific Processes in Nucleus Pulposus and Annulus Fibrosus During Intervertebral Disc Degeneration. Adv Sci (Weinh).

[2] Burt KG, Kim MKM, Viola DC, Abraham AC, Chahine NO (2024). Nuclear factor κB overactivation in the intervertebral disc leads to macrophage recruitment and severe disc degeneration. Sci Adv.

[3] Evans AD, Pournoori N, Saksala E, Oommen OP (2024). Glycosaminoglycans' for brain health: Harnessing glycosaminoglycan based biomaterials for treating central nervous system diseases and in-vitro modeling. Biomaterials.

[4] Zheng D, Chen W, Chen T, Chen X, Liang J, Chen H (2024). Hydrogen Ion Capturing Hydrogel Microspheres for Reversing Inflammaging. Adv Mater.

[5] Genedy HH, Humbert P, Laoulaou B, Le Moal B, Fusellier M, Passirani C (2024). MicroRNA-targeting nanomedicines for the treatment of intervertebral disc degeneration. Adv Drug Deliv Rev.

[6] Novais EJ, Narayanan R, Canseco JA, van de Wetering K, Kepler CK, Hilibrand AS (2024). A new perspective on intervertebral disc calcification-from bench to bedside. Bone Res.

[7] Ohnishi T, Tran V, Sao K, Ramteke P, Querido W, Barve RA (2023). Loss of function mutation in Ank causes aberrant mineralization and acquisition of osteoblast-like-phenotype by the cells of the intervertebral disc. Cell Death Dis.

[8] Sun K, Yan C, Dai X, Shi Y, Li F, Chen L (2024). Catalytic Nanodots-Driven Pyroptosis Suppression in Nucleus Pulposus for Antioxidant Intervention of Intervertebral Disc Degeneration. Adv Mater.

[9] Gao B, Jiang B, Xing W, Xie Z, Luo Z, Zou W (2022). Discovery and Application of Postnatal Nucleus Pulposus Progenitors Essential for Intervertebral Disc Homeostasis and Degeneration. Adv Sci (Weinh).

[10] Nakielski P, Rybak D, Jezierska-Woźniak K, Rinoldi C, Sinderewicz E, Staszkiewicz-Chodor J (2023). Minimally Invasive Intradiscal Delivery of BM-MSCs via Fibrous Microscaffold Carriers. ACS Appl Mater Interfaces.

[11] Kelly K, Bloor AJC, Griffin JE, Radia R, Yeung DT, Rasko JEJ (2024). Two-year safety outcomes of iPS cell-derived mesenchymal stromal cells in acute steroid-resistant graft-versus-host disease. Nat Med.

[12] Lee SB, Abdal Dayem A, Kmiecik S, Lim KM, Seo DS, Kim H-T (2024). Efficient improvement of the proliferation, differentiation, and anti-arthritic capacity of mesenchymal stem cells by simply culturing on the immobilized FGF2 derived peptide, 44-ERGVVSIKGV-53. J Adv Res.

[13] Gomez-Ruiz V, Blanco JF, Villarón EM, Fidalgo H, López-Parra M, Sánchez-Guijo F (2023). Autologous mesenchymal stem cell transplantation for spinal fusion: 10 years follow-up of a phase I/II clinical trial. Stem Cell Res Ther.

[14] Peng Y, Chen X, Zhang Q, Liu S, Wu W, Li K (2024). Enzymatically Bioactive Nucleus Pulposus Matrix Hydrogel Microspheres for Exogenous Stem Cells Therapy and Endogenous Repair Strategy to Achieve Disc Regeneration. Adv Sci (Weinh).

[15] Sun J, Yang F, Wang L, Yu H, Yang Z, Wei J (2023). Delivery of coenzyme Q10 loaded micelle targets mitochondrial ROS and enhances efficiency of mesenchymal stem cell therapy in intervertebral disc degeneration. Bioact Mater.

[16] Ukeba D, Yamada K, Suyama T, Lebl DR, Tsujimoto T, Nonoyama T (2022). Combination of ultra-purified stem cells with an in situ-forming bioresorbable gel enhances intervertebral disc regeneration. EBioMedicine.

[17] Peng Y, Qing X, Lin H, Huang D, Li J, Tian S (2021). Decellularized Disc Hydrogels for hBMSCs tissue-specific differentiation and tissue regeneration. Bioact Mater.

[18] Shi M, Zhao Y, Sun Y, Xin D, Xu W, Zhou B (2021). Therapeutic effect of co-culture of rat bone marrow mesenchymal stem cells and degenerated nucleus pulposus cells on intervertebral disc degeneration. Spine J.

[19] Feng G, Zhang Z, Dang M, Rambhia KJ, Ma PX (2020). Nanofibrous spongy microspheres to deliver rabbit mesenchymal stem cells and anti-miR-199a to regenerate nucleus pulposus and prevent calcification. Biomaterials.

[20] Huang G, Shen H, Xu K, Shen Y, Jiale J, Chu G (2024). Single-Cell Microgel Encapsulation Improves the Therapeutic Efficacy of Mesenchymal Stem Cells in Treating Intervertebral Disc Degeneration via Inhibiting Pyroptosis. Research (Wash D C).

[21] Liu Y, Li L, Li X, Cherif H, Jiang S, Ghezelbash F (2024). Viscoelastic hydrogels regulate adipose-derived mesenchymal stem cells for nucleus pulposus regeneration. Acta Biomater.

[22] Lin C-L, Su Y-W, Chen Y-W, Kuo C-H, Tu T-Y, Tsai J-C (2024). BMSC loaded photo-crosslinked hyaluronic acid/collagen hydrogel incorporating FG4592 for enhanced cell proliferation and nucleus pulposus differentiation. Int J Biol Macromol.

[23] Wang Z, Yang H, Xu X, Hu H, Bai Y, Hai J (2023). Ion elemental-optimized layered double hydroxide nanoparticles promote chondrogenic differentiation and intervertebral disc regeneration of mesenchymal stem cells through focal adhesion signaling pathway. Bioact Mater.

[24] Zhao D-W, Cheng Q, Geng H, Liu J, Zhang Y, Cui J (2024). Decoding Macrophage Subtypes to Engineer Modulating Hydrogels for the Alleviation of Intervertebral Disk Degeneration. Adv Sci (Weinh).

[25] Wang T, Zhao H, Jing S, Fan Y, Sheng G, Ding Q (2023). Magnetofection of miR-21 promoted by electromagnetic field and iron oxide nanoparticles via the p38 MAPK pathway contributes to osteogenesis and angiogenesis for intervertebral fusion. J Nanobiotechnology.

[26] Xu Y, Fan P, Xu X, Liu L, Zhang L, Li X, Wang J, Tao Y, Li X, Xu D, Wang X, Zhou Y, Wang Y (2023). Tert-butyl hydroperoxide induces ferroptosis of bone mesenchymal stem cells by repressing the prominin2/BACH1/ROS axis. Am J Physiol Cell Physiol.

[27] Hu G, Cui Z, Chen X, Sun F, Li T, Li C (2023). Suppressing Mesenchymal Stromal Cell Ferroptosis Via Targeting a Metabolism-Epigenetics Axis Corrects their Poor Retention and Insufficient Healing Benefits in the Injured Liver Milieu. Adv Sci (Weinh).

[28] Fu Y, Sui B, Xiang L, Yan X, Wu D, Shi S (2021). Emerging understanding of apoptosis in mediating mesenchymal stem cell therapy. Cell Death Dis.

[29] Zhang X, Yang J, Ma S, Gao X, Wang G, Sun Y (2024). Functional diversity of apoptotic vesicle subpopulations from bone marrow mesenchymal stem cells in tissue regeneration. J Extracell Vesicles.

[30] Wu X, Zhang F, Mao X, Xu F, Ding X, Sun X (2024). The mechanism of adipose mesenchymal stem cells to stabilize the immune microenvironment of pelvic floor injury by regulating pyroptosis and promoting tissue repair. Mater Today Bio.

[31] Rojas-Rivera D, Beltrán S, Muñoz-Carvajal F, Ahumada-Montalva P, Abarzúa L, Gomez L (2024). The autophagy protein RUBCNL/PACER represses RIPK1 kinase-dependent apoptosis and necroptosis. Autophagy.

[32] Pang SHM, D'Rozario J, Mendonca S, Bhuvan T, Payne NL, Zheng D (2021). Mesenchymal stromal cell apoptosis is required for their therapeutic function. Nat Commun.

[33] Ying Y, Huang Z, Tu Y, Wu Q, Li Z, Zhang Y (2023). A shear-thinning, ROS-scavenging hydrogel combined with dental pulp stem cells promotes spinal cord repair by inhibiting ferroptosis. Bioact Mater.

[34] Li Y, Cai Z, Ma W, Bai L, Luo E, Lin Y (2024). A DNA tetrahedron-based ferroptosis-suppressing nanoparticle: superior delivery of curcumin and alleviation of diabetic osteoporosis. Bone Res.

[35] Yuan K, Yang Y, Lin Y, Zhou F, Huang K, Yang S (2024). Targeting Bacteria-Induced Ferroptosis of Bone Marrow Mesenchymal Stem Cells to Promote the Repair of Infected Bone Defects. Adv Sci (Weinh).

[36] Huang L, Zhang S, Bian M, Xiang X, Xiao L, Wang J (2024). Injectable, anti-collapse, adhesive, plastic and bioactive bone graft substitute promotes bone regeneration by moderating oxidative stress in osteoporotic bone defect. Acta Biomater.

[37] Jing Z, Li Y, Zhang H, Chen T, Yu J, Xu X (2023). Tobacco toxins induce osteoporosis through ferroptosis. Redox Biol.

[38] Schwab A, Rao Z, Zhang J, Gollowitzer A, Siebenkäs K, Bindel N (2024). Zeb1 mediates EMT/plasticity-associated ferroptosis sensitivity in cancer cells by regulating lipogenic enzyme expression and phospholipid composition. Nat Cell Biol.

[39] Ru Q, Li Y, Chen L, Wu Y, Min J, Wang F (2024). Iron homeostasis and ferroptosis in human diseases: mechanisms and therapeutic prospects. Signal Transduct Target Ther.

[40] Jiang X, Peng Q, Peng M, Oyang L, Wang H, Liu Q (2024). Cellular metabolism: A key player in cancer ferroptosis. Cancer Commun (Lond).

[41] Wang W, Jing X, Du T, Ren J, Liu X, Chen F (2022). Iron overload promotes intervertebral disc degeneration via inducing oxidative stress and ferroptosis in endplate chondrocytes. Free Radic Biol Med.

[42] Yang X, Chen Y, Guo J, Li J, Zhang P, Yang H (2023). Polydopamine Nanoparticles Targeting Ferroptosis Mitigate Intervertebral Disc Degeneration Via Reactive Oxygen Species Depletion, Iron Ions Chelation, and GPX4 Ubiquitination Suppression. Adv Sci (Weinh).

[43] Xu Y, Fan P, Liu L, Xuanfei XU, Zhang L, Wang J (2023). Novel perspective in transplantation therapy of mesenchymal stem cells: targeting the ferroptosis pathway. J Zhejiang Univ Sci B.

[44] Yuan Q, Du L, Xu H, Zhang K, Li Q, Zhang H (2022). Autologous Mesenchymal Stromal Cells Combined with Gelatin Sponge for Repair Intervertebral Disc Defect after Discectomy: A Preclinical Study in a Goat Model. Front Biosci (Landmark Ed).

[45] Blanco JF, Villarón EM, Pescador D, da Casa C, Gómez V, Redondo AM (2019). Autologous mesenchymal stromal cells embedded in tricalcium phosphate for posterolateral spinal fusion: results of a prospective phase I/II clinical trial with long-term follow-up. Stem Cell Res Ther.

[46] Henriksson HB, Papadimitriou N, Hingert D, Baranto A, Lindahl A, Brisby H (2019). The Traceability of Mesenchymal Stromal Cells After Injection Into Degenerated Discs in Patients with Low Back Pain. Stem Cells Dev.

[47] Noriega DC, Ardura F, Hernández-Ramajo R, Martín-Ferrero MÁ, Sánchez-Lite I, Toribio B (2021). Treatment of Degenerative Disc Disease With Allogeneic Mesenchymal Stem Cells: Long-term Follow-up Results. Transplantation.

[48] Amirdelfan K, Bae H, McJunkin T, DePalma M, Kim K, Beckworth WJ (2021). Allogeneic mesenchymal precursor cells treatment for chronic low back pain associated with degenerative disc disease: a prospective randomized, placebo-controlled 36-month study of safety and efficacy. Spine J.

[49] Atluri S, Murphy MB, Dragella R, Herrera J, Boachie-Adjei K, Bhati S (2022). Evaluation of the Effectiveness of Autologous Bone Marrow Mesenchymal Stem Cells in the Treatment of Chronic Low Back Pain Due to Severe Lumbar Spinal Degeneration: A 12-Month, Open-Label, Prospective Controlled Trial. Pain Physician.

[50] Zhang J, Sun T, Zhang W, Yang M, Li Z (2022). Autologous cultured adipose derived mesenchymal stem cells combined with hyaluronic acid hydrogel in the treatment of discogenic low back pain: a study protocol for a phase II randomised controlled trial. BMJ Open.

[51] Sudo H, Miyakoshi T, Watanabe Y, Ito YM, Kahata K, Tha KK (2023). Protocol for treating lumbar spinal canal stenosis with a combination of ultrapurified, allogenic bone marrow-derived mesenchymal stem cells and in situ-forming gel: a multicentre, prospective, double-blind randomised controlled trial. BMJ Open.

[52] Lewandrowski K-U, Dowling A, Vera JC, Leon JFR, Telfeian AE, Lorio MP (2023). Pain Relief After Allogenic Stem Cell Disc Therapy. Pain Physician.

[53] Lee DH, Park K-S, Shin HE, Kim SB, Choi H, An SB (2023). Safety and Feasibility of Intradiscal Administration of Matrilin-3-Primed Adipose-Derived Mesenchymal Stromal Cell Spheroids for Chronic Discogenic Low Back Pain: Phase 1 Clinical Trial. Int J Mol Sci.

[54] Gopalarethinam J, Nair AP, Iyer M, Vellingiri B, Subramaniam MD (2023). Advantages of mesenchymal stem cell over the other stem cells. Acta Histochem.

[55] Yang W, Jin M, Gu Y, Zhao X, Zhu L, He S (2024). Intracellular osteopontin potentiates the immunosuppressive activity of mesenchymal stromal cells. Stem Cell Res Ther.

[56] Wang Y, Fang J, Liu B, Shao C, Shi Y (2022). Reciprocal regulation of mesenchymal stem cells and immune responses. Cell Stem Cell.

[57] Dave M, Dev A, Somoza RA, Zhao N, Viswanath S, Mina PR (2024). MSCs mediate long-term efficacy in a Crohn's disease model by sustained anti-inflammatory macrophage programming via efferocytosis. NPJ Regen Med.

[58] Zou Z, Lin Z, Wu C, Tan J, Zhang J, Peng Y (2023). Micro-Engineered Organoid-on-a-Chip Based on Mesenchymal Stromal Cells to Predict Immunotherapy Responses of HCC Patients. Adv Sci (Weinh).

[59] Harrell CR, Volarevic A, Djonov VG, Jovicic N, Volarevic V (2021). Mesenchymal Stem Cell: A Friend or Foe in Anti-Tumor Immunity. Int J Mol Sci.

[60] Liu H, Zhang X, Zhang M, Zhang S, Li J, Zhang Y (2024). Mesenchymal Stem Cell Derived Exosomes Repair Uterine Injury by Targeting Transforming Growth Factor-β Signaling. ACS Nano.

[61] Sun Y, Liu Q, Qin Y, Xu Y, Zhao J, Xie Y (2024). Exosomes derived from CD271+CD56+ bone marrow mesenchymal stem cell subpopoulation identified by single-cell RNA sequencing promote axon regeneration after spinal cord injury. Theranostics.

[62] Tan F, Li X, Wang Z, Li J, Shahzad K, Zheng J (2024). Clinical applications of stem cell-derived exosomes. Signal Transduct Target Ther.

[63] Lian M, Qiao Z, Qiao S, Zhang X, Lin J, Xu R (2024). Nerve Growth Factor-Preconditioned Mesenchymal Stem Cell-Derived Exosome-Functionalized 3D-Printed Hierarchical Porous Scaffolds with Neuro-Promotive Properties for Enhancing Innervated Bone Regeneration. ACS Nano.

[64] Tan SHS, Wong JRY, Sim SJY, Tjio CKE, Wong KL, Chew JRJ (2020). Mesenchymal stem cell exosomes in bone regenerative strategies-a systematic review of preclinical studies. Mater Today Bio.

[65] Tieu A, Lalu MM, Slobodian M, Gnyra C, Fergusson DA, Montroy J (2020). An Analysis of Mesenchymal Stem Cell-Derived Extracellular Vesicles for Preclinical Use. ACS Nano.

[66] Shi P, Gao H, Cheng Z, Zhao K, Chen Y, Chen X (2024). Static magnetic field-modulated mesenchymal stem cell-derived mitochondria-containing microvesicles for enhanced intervertebral disc degeneration therapy. J Nanobiotechnology.

[67] Dixon SJ, Olzmann JA (2024). The cell biology of ferroptosis. Nat Rev Mol Cell Biol.

[68] Nakamura T, Conrad M (2024). Exploiting ferroptosis vulnerabilities in cancer. Nat Cell Biol.

[69] Maremonti F, Tonnus W, Gavali S, Bornstein S, Shah A, Giacca M (2024). Ferroptosis-based advanced therapies as treatment approaches for metabolic and cardiovascular diseases. Cell Death Differ.

[70] Morgan PK, Pernes G, Huynh K, Giles C, Paul S, Smith AAT (2024). A lipid atlas of human and mouse immune cells provides insights into ferroptosis susceptibility. Nat Cell Biol.

[71] Zhou S, Liu J, Wan A, Zhang Y, Qi X (2024). Epigenetic regulation of diverse cell death modalities in cancer: a focus on pyroptosis, ferroptosis, cuproptosis, and disulfidptosis. J Hematol Oncol.

[72] Weichhart T (2024). Transferrin: the iron transporter takes control. Blood.

[73] Galy B, Conrad M, Muckenthaler M (2024). Mechanisms controlling cellular and systemic iron homeostasis. Nat Rev Mol Cell Biol.

[74] Shagidov D, Guttmann-Raviv N, Cunat S, Frech L, Giansily-Blaizot M, Ghatpande N (2024). A newly identified ferritin L-subunit variant results in increased proteasomal subunit degradation, impaired complex assembly, and severe hypoferritinemia. Am J Hematol.

[75] Hoelzgen F, Nguyen TTP, Klukin E, Boumaiza M, Srivastava AK, Kim EY (2024). Structural basis for the intracellular regulation of ferritin degradation. Nat Commun.

[76] Co HKC, Wu C-C, Lee Y-C, Chen S-H (2024). Emergence of large-scale cell death through ferroptotic trigger waves. Nature.

[77] Balakrishnan M, Kenworthy AK (2024). Lipid Peroxidation Drives Liquid-Liquid Phase Separation and Disrupts Raft Protein Partitioning in Biological Membranes. J Am Chem Soc.

[78] Perluigi M, Di Domenico F, Butterfield DA (2024). Oxidative damage in neurodegeneration: roles in the pathogenesis and progression of Alzheimer disease. Physiol Rev.

[79] Hines MR, Gomez-Contreras PC, Liman S, Wilson AM, Lu KJ, O'Neill JA (2024). A reciprocal relationship between mitochondria and lipid peroxidation determines the chondrocyte intracellular redox environment. Redox Biol.

[80] Kanaan MN, Pileggi CA, Karam CY, Kennedy LS, Fong-McMaster C, Cuperlovic-Culf M (2024). Cystine/glutamate antiporter xCT controls skeletal muscle glutathione redox, bioenergetics and differentiation. Redox Biol.

[81] Ahola S, Langer T (2024). Ferroptosis in mitochondrial cardiomyopathy. Trends Cell Biol.

[82] Zhang H, Chen H, Lu L, Wang H, Zhao Y, Chai R (2024). Natural Multifunctional Silk Microcarriers for Noise-Induced Hearing Loss Therapy. Adv Sci (Weinh).

[83] Huang Y, Liu J, He J, Tan F, Lu M, Yuan F (2023). Curcumin preconditioning enhances the neuroprotective effects of olfactory mucosa-derived mesenchymal stem cells on experimental intracerebral hemorrhage. Heliyon.

[84] Xu C-Y, Xu C, Xu Y-N, Du S-Q, Dai Z-H, Jin S-Q (2024). Poliumoside protects against type 2 diabetes-related osteoporosis by suppressing ferroptosis via activation of the Nrf2/GPX4 pathway. Phytomedicine.

[85] Huang L, Wang J, Yu J, Bian M, Xiang X, Han G (2024). Picein alleviates oxidative stress and promotes bone regeneration in osteoporotic bone defect by inhibiting ferroptosis via Nrf2/HO-1/GPX4 pathway. Environ Toxicol.

[86] Yang A, Wang L, Jiang K, Lei L, Li H (2021). Nuclear receptor coactivator 4-mediated ferritinophagy drives proliferation of dental pulp stem cells in hypoxia. Biochem Biophys Res Commun.

[87] Liu J, Ren Z, Yang L, Zhu L, Li Y, Bie C (2022). The NSUN5-FTH1/FTL pathway mediates ferroptosis in bone marrow-derived mesenchymal stem cells. Cell Death Discov.

[88] Kannan M, Sil S, Oladapo A, Thangaraj A, Periyasamy P, Buch S (2023). HIV-1 Tat-mediated microglial ferroptosis involves the miR-204-ACSL4 signaling axis. Redox Biol.

[89] Hopfner U, Maan ZN, Hu MS, Aitzetmüller MM, Zaussinger M, Kirsch M (2020). Deferoxamine enhances the regenerative potential of diabetic Adipose Derived Stem Cells. J Plast Reconstr Aesthet Surg.

[90] Khoshlahni N, Sagha M, Mirzapour T, Zarif MN, Mohammadzadeh-Vardin M (2020). Iron depletion with deferoxamine protects bone marrow-derived mesenchymal stem cells against oxidative stress-induced apoptosis. Cell Stress Chaperones.

[91] Li X, Cheng Y, Yang Z, Ji Q, Huan M, Ye W (2024). Glioma-targeted oxaliplatin/ferritin clathrate reversing the immunosuppressive microenvironment through hijacking Fe2+ and boosting Fenton reaction. J Nanobiotechnology.

[92] Lazourgui MA, El-Aoufi S, Labsi M, Maouche B (2016). Coenzyme Q10 Supplementation Prevents Iron Overload While Improving Glycaemic Control and Antioxidant Protection in Insulin-Resistant Psammomys obesus. Biol Trace Elem Res.

[93] Bersuker K, Hendricks JM, Li Z, Magtanong L, Ford B, Tang PH (2019). The CoQ oxidoreductase FSP1 acts parallel to GPX4 to inhibit ferroptosis. Nature.

[94] Peng Z, Ding Y-N, Yang Z-M, Li X-J, Zhuang Z, Lu Y (2024). Neuron-targeted liposomal coenzyme Q10 attenuates neuronal ferroptosis after subarachnoid hemorrhage by activating the ferroptosis suppressor protein 1/coenzyme Q10 system. Acta Biomater.

[95] Ming T, Wu Y, Huan H, Jiang Q, Su C, Lu C (2021). Integrative proteomics and metabolomics profiling of the protective effects of Phascolosoma esculent ferritin on BMSCs in Cd(II) injury. Ecotoxicol Environ Saf.

[96] Ren E, Chen H, Qin Z, Guan S, Jiang L, Pang X (2022). Harnessing Bifunctional Ferritin with Kartogenin Loading for Mesenchymal Stem Cell Capture and Enhancing Chondrogenesis in Cartilage Regeneration. Adv Healthc Mater.

[97] Chen X, Zhang A, Zhao K, Gao H, Shi P, Chen Y (2024). The role of oxidative stress in intervertebral disc degeneration: Mechanisms and therapeutic implications. Ageing Res Rev.

[98] Tamagawa S, Sakai D, Nojiri H, Nakamura Y, Warita T, Matsushita E (2024). SOD2 orchestrates redox homeostasis in intervertebral discs: A novel insight into oxidative stress-mediated degeneration and therapeutic potential. Redox Biol.

[99] Yang L, Bhujel B, Hou Y, Luo J, An SB, Han I (2023). Effective Modulation of Inflammation and Oxidative Stress for Enhanced Regeneration of Intervertebral Discs Using 3D Porous Hybrid Protein Nanoscaffold. Adv Mater.

[100] Chen Q, Qian Q, Xu H, Zhou H, Chen L, Shao N (2024). Mitochondrial-Targeted Metal-Phenolic Nanoparticles to Attenuate Intervertebral Disc Degeneration: Alleviating Oxidative Stress and Mitochondrial Dysfunction. ACS Nano.

[101] Bu W, Shi Y, Huang X, Wu S, Jiang L, Pan C (2024). Rescue of nucleus pulposus cells from an oxidative stress microenvironment via glutathione-derived carbon dots to alleviate intervertebral disc degeneration. J Nanobiotechnology.

[102] Wang W, Liu L, Ma W, Zhao L, Huang L, Zhou D (2024). An anti-senescence hydrogel with pH-responsive drug release for mitigating intervertebral disc degeneration and low back pain. Bioact Mater.

[103] Bell HN, Stockwell BR, Zou W (2024). Ironing out the role of ferroptosis in immunity. Immunity.

[104] Liang FG, Zandkarimi F, Lee J, Axelrod JL, Pekson R, Yoon Y (2024). OPA1 promotes ferroptosis by augmenting mitochondrial ROS and suppressing an integrated stress response. Mol Cell.

[105] Bruedigam C, Porter AH, Song A, Vroeg In de Wei G, Stoll T, Straube J (2024). Imetelstat-mediated alterations in fatty acid metabolism to induce ferroptosis as a therapeutic strategy for acute myeloid leukemia. Nat Cancer.

[106] Tak J, Joo MS, Kim YS, Park HW, Lee CH, Park G-C (2024). Dual regulation of NEMO by Nrf2 and miR-125a inhibits ferroptosis and protects liver from endoplasmic reticulum stress-induced injury. Theranostics.

[107] Sánchez-Ortega M, Garrido A, Cirauqui C, Sanz-Gonzalez L, Hernández MC, González-García A (2024). A potential therapeutic strategy based on acute oxidative stress induction for wild-type NRF2/KEAP1 lung squamous cell carcinoma. Redox Biol.

[108] Park MW, Cha HW, Kim J, Kim JH, Yang H, Yoon S (2021). NOX4 promotes ferroptosis of astrocytes by oxidative stress-induced lipid peroxidation via the impairment of mitochondrial metabolism in Alzheimer's diseases. Redox Biol.

[109] Li X, Zeng J, Liu Y, Liang M, Liu Q, Li Z (2020). Inhibitory Effect and Mechanism of Action of Quercetin and Quercetin Diels-Alder anti-Dimer on Erastin-Induced Ferroptosis in Bone Marrow-Derived Mesenchymal Stem Cells. Antioxidants (Basel).

[110] Wang H, Wei X, Liu L, Zhang J, Li H (2024). Suppression of A-to-I RNA-editing enzyme ADAR1 sensitizes hepatocellular carcinoma cells to oxidative stress through regulating Keap1/Nrf2 pathway. Exp Hematol Oncol.

[111] Zhuang H, Ren X, Zhang Y, Li H, Zhou P (2024). β-Hydroxybutyrate enhances chondrocyte mitophagy and reduces cartilage degeneration in osteoarthritis via the HCAR2/AMPK/PINK1/Parkin pathway. Aging Cell.

[112] Li X, Wang Q, Xu C, Zhang L, Zhou J, Lv J (2023). Ferroptosis Inducers Kill Mesenchymal Stem Cells Affected by Neuroblastoma. Cancers (Basel).

[113] Zhang H, Pan J, Huang S, Chen X, Chang ACY, Wang C (2024). Hydrogen sulfide protects cardiomyocytes from doxorubicin-induced ferroptosis through the SLC7A11/GSH/GPx4 pathway by Keap1 S-sulfhydration and Nrf2 activation. Redox Biol.

[114] Ma L, Chen C, Zhao C, Li T, Ma L, Jiang J (2024). Targeting carnitine palmitoyl transferase 1A (CPT1A) induces ferroptosis and synergizes with immunotherapy in lung cancer. Signal Transduct Target Ther.

[115] Bhat KP, Vijay J, Vilas CK, Asundi J, Zou J, Lau T (2024). CRISPR activation screens identify the SWI/SNF ATPases as suppressors of ferroptosis. Cell Rep.

[116] Sastre J, Pérez S, Sabater L, Rius-Pérez S (2025). REDOX SIGNALLING IN THE PANCREAS IN HEALTH AND DISEASE. Physiol Rev.

[117] Koppula P, Lei G, Zhang Y, Yan Y, Mao C, Kondiparthi L (2022). A targetable CoQ-FSP1 axis drives ferroptosis- and radiation-resistance in KEAP1 inactive lung cancers. Nat Commun.

[118] Huang L, Bian M, Lu S, Wang J, Yu J, Jiang L (2023). Engeletin alleviates erastin-induced oxidative stress and protects against ferroptosis via Nrf2/Keap1 pathway in bone marrow mesenchymal stem cells. Tissue Cell.

[119] Torres-Torres J, Espino-Y-Sosa S, Martinez-Portilla R, Borboa-Olivares H, Estrada-Gutierrez G, Acevedo-Gallegos S (2024). A Narrative Review on the Pathophysiology of Preeclampsia. Int J Mol Sci.

[120] Kolluru GK, Bir SC, Yuan S, Shen X, Pardue S, Wang R (2015). Cystathionine γ-lyase regulates arteriogenesis through NO-dependent monocyte recruitment. Cardiovasc Res.

[121] Hassan N, Yi H, Malik B, Gaspard-Boulinc L, Samaraweera SE, Casolari DA (2024). Loss of the stress sensor GADD45A promotes stem cell activity and ferroptosis resistance in LGR4/HOXA9-dependent AML. Blood.

[122] Hu B, Zhang X-X, Zhang T, Yu W-C (2023). Dissecting molecular mechanisms underlying ferroptosis in human umbilical cord mesenchymal stem cells: Role of cystathionine γ-lyase/hydrogen sulfide pathway. World J Stem Cells.

[123] Cui P, Sheng Y, Wu C, He D (2024). Puerarin modulates proliferation, inflammation and ECM metabolism in human nucleus pulposus mesenchymal stem cells via the lncRNA LINC01535. Heliyon.

[124] Wang F, Guo K, Nan L, Wang S, Lu J, Wang Q (2023). Kartogenin-loaded hydrogel promotes intervertebral disc repair via protecting MSCs against reactive oxygen species microenvironment by Nrf2/TXNIP/NLRP3 axis. Free Radic Biol Med.

[125] Huang Z, Chen G, Wu H, Huang X, Xu R, Deng F (2023). Ebselen restores peri-implantitis-induced osteogenic inhibition via suppressing BMSCs ferroptosis. Exp Cell Res.

[126] Chen B, Li X, Liu J, Li Y, Dai W, Chen Y (2021). Ferroptosis-Inhibitory Effect and Possible Mechanisms of Ellagitannin Geraniin. ChemistryOpen.

[127] Liu J, Pang S-Y, Zhou S-Y, He Q-Y, Zhao R-Y, Qu Y (2024). Lipocalin-2 aggravates blood-brain barrier dysfunction after intravenous thrombolysis by promoting endothelial cell ferroptosis via regulating the HMGB1/Nrf2/HO-1 pathway. Redox Biol.

[128] Vukelić D, Baralić K, Marić Đ, Đukic-Ćosić D, Bulat Z, Panieri E (2024). Hepato-renal toxicity of low dose metal(oid)s mixture in real-life risk simulation in rats: Effects on Nrf2/HO-1 signalling and redox status. Sci Total Environ.

[129] Jegadheeshwari S, Santhi JJ, Velayutham M, Issac PK, Kesavan M (2024). DbGTi protein attenuates chromium (VI)-induced oxidative stress via activation of the Nrf2/HO-1 signalling pathway in zebrafish (Danio rerio) larval model. Int J Biol Macromol.

[130] Morgenstern C, Lastres-Becker I, Demirdöğen BC, Costa VM, Daiber A, Foresti R (2024). Biomarkers of NRF2 signalling: Current status and future challenges. Redox Biol.

[131] Patibandla C, van Aalten L, Dinkova-Kostova AT, Honda T, Cuadrado A, Fernández-Ginés R (2024). Inhibition of glycogen synthase kinase-3 enhances NRF2 protein stability, nuclear localisation and target gene transcription in pancreatic beta cells. Redox Biol.

[132] Zeng Y-L, Liu L-Y, Ma T-Z, Liu Y, Liu B, Liu W (2024). Iridium(III) Photosensitizers Induce Simultaneous Pyroptosis and Ferroptosis for Multi-Network Synergistic Tumor Immunotherapy. Angew Chem Int Ed Engl.

[133] Wang H, Yu X, Liu D, Qiao Y, Huo J, Pan S (2024). VDR Activation Attenuates Renal Tubular Epithelial Cell Ferroptosis by Regulating Nrf2/HO-1 Signaling Pathway in Diabetic Nephropathy. Adv Sci (Weinh).

[134] Zhang Z, Qin F, Feng Y, Zhang S, Xie C, Huang H (2022). Icariin regulates stem cell migration for endogenous repair of intervertebral disc degeneration by increasing the expression of chemotactic cytokines. BMC Complement Med Ther.

[135] Fang Y, Hu J, Zou Y, Wang Z, Ye Y, Zhang C (2024). Neochlorogenic Acid Combined with Bone Marrow Mesenchymal Stem Cells Encapsulated into GelMA Hydrogel for Transplantation to Repair Intervertebral Disk Degeneration. Biomacromolecules.

[136] Hu J, Li C, Jin S, Ye Y, Fang Y, Xu P (2022). Salvianolic acid B combined with bone marrow mesenchymal stem cells piggybacked on HAMA hydrogel re-transplantation improves intervertebral disc degeneration. Front Bioeng Biotechnol.

[137] Xia Y, Zhang H, Wang H, Wang Q, Zhu P, Gu Y (2022). Identification and validation of ferroptosis key genes in bone mesenchymal stromal cells of primary osteoporosis based on bioinformatics analysis. Front Endocrinol (Lausanne).

[138] Wang D, Zhang H, Liao X, Li J, Zeng J, Wang Y (2024). Oral administration of Robinia pseudoacacia L. flower exosome-like nanoparticles attenuates gastric and small intestinal mucosal ferroptosis caused by hypoxia through inhibiting HIF-1α- and HIF-2α-mediated lipid peroxidation. J Nanobiotechnology.

[139] Yang Z, Su W, Wei X, Qu S, Zhao D, Zhou J (2023). HIF-1α drives resistance to ferroptosis in solid tumors by promoting lactate production and activating SLC1A1. Cell Rep.

[140] Li M, Li L, Cheng X, Li L, Tu K (2023). Hypoxia promotes the growth and metastasis of ovarian cancer cells by suppressing ferroptosis via upregulating SLC2A12. Exp Cell Res.

[141] Yu C, Li D, Wang C, Xia K, Wang J, Zhou X (2021). Injectable kartogenin and apocynin loaded micelle enhances the alleviation of intervertebral disc degeneration by adipose-derived stem cell. Bioact Mater.

[142] Huang X, Chen D, Liang C, Shi K, Zhou X, Zhang Y (2023). Swelling-Mediated Mechanical Stimulation Regulates Differentiation of Adipose-Derived Mesenchymal Stem Cells for Intervertebral Disc Repair Using Injectable UCST Microgels. Adv Healthc Mater.

[143] Wu J, Yu L, Liu Y, Xiao B, Ye X, Zhao H (2023). Hypoxia regulates adipose mesenchymal stem cells proliferation, migration, and nucleus pulposus-like differentiation by regulating endoplasmic reticulum stress via the HIF-1α pathway. J Orthop Surg Res.

[144] He R, Wang Z, Cui M, Liu S, Wu W, Chen M (2021). HIF1A Alleviates compression-induced apoptosis of nucleus pulposus derived stem cells via upregulating autophagy. Autophagy.

[145] Chen BY, Pathak JL, Lin HY, Guo WQ, Chen WJ, Luo G (2024). Inflammation Triggers Chondrocyte Ferroptosis in TMJOA via HIF-1α/TFRC. J Dent Res.

[146] Lin Z, Song J, Gao Y, Huang S, Dou R, Zhong P (2022). Hypoxia-induced HIF-1α/lncRNA-PMAN inhibits ferroptosis by promoting the cytoplasmic translocation of ELAVL1 in peritoneal dissemination from gastric cancer. Redox Biol.

[147] Rakshe PS, Dutta BJ, Chib S, Maurya N, Singh S (2024). Unveiling the interplay of AMPK/SIRT1/PGC-1α axis in brain health: Promising targets against aging and NDDs. Ageing Res Rev.

[148] Thapa R, Moglad E, Afzal M, Gupta G, Bhat AA, Hassan Almalki W (2024). The role of sirtuin 1 in ageing and neurodegenerative disease: A molecular perspective. Ageing Res Rev.

[149] Sanz-Alcázar A, Portillo-Carrasquer M, Delaspre F, Pazos-Gil M, Tamarit J, Ros J (2024). Deciphering the ferroptosis pathways in dorsal root ganglia of Friedreich ataxia models. The role of LKB1/AMPK, KEAP1, and GSK3β in the impairment of the NRF2 response. Redox Biol.

[150] Zhao W-J, Liu X, Hu M, Zhang Y, Shi P-Z, Wang J-W (2023). Quercetin ameliorates oxidative stress-induced senescence in rat nucleus pulposus-derived mesenchymal stem cells via the miR-34a-5p/SIRT1 axis. World J Stem Cells.

[151] Zhou Q, Zhu C, Xuan A, Zhang J, Zhu Z, Tang L (2023). Fisetin regulates the biological effects of rat nucleus pulposus mesenchymal stem cells under oxidative stress by sirtuin-1 pathway. Immun Inflamm Dis.

[152] Wang P, Yang C, Lu J, Ren Y, Goltzman D, Miao D (2023). Sirt1 protects against intervertebral disc degeneration induced by 1,25-dihydroxyvitamin D insufficiency in mice by inhibiting the NF-κB inflammatory pathway. J Orthop Translat.

[153] Stouth DW, vanLieshout TL, Mikhail AI, Ng SY, Raziee R, Edgett BA (2024). CARM1 drives mitophagy and autophagy flux during fasting-induced skeletal muscle atrophy. Autophagy.

[154] Xie T, Pan R, Huang W, Dong S, Wu S, Ye Y (2023). Myricetin alleviates H2O2-induced senescence and apoptosis in rat nucleus pulposus-derived mesenchymal stem cells. Folia Histochem Cytobiol.

[155] Weng J, Liu Q, Li C, Feng Y, Chang Q, Xie M (2024). TRPA1-PI3K/Akt-OPA1-ferroptosis axis in ozone-induced bronchial epithelial cell and lung injury. Sci Total Environ.

[156] Zheng X, Zhang Y, Zhang L, Yang T, Zhang F, Wang X (2024). Taurolithocholic acid protects against viral haemorrhagic fever via inhibition of ferroptosis. Nat Microbiol.

[157] Tani T, Oikawa M, Misaka T, Ishida T, Takeishi Y (2024). Heart Failure Post-Myocardial Infarction Promotes Mammary Tumor Growth Through the NGF-TRKA Pathway. JACC CardioOncol.

[158] Abdullah KM, Sharma G, Qais FA, Khan I, Takkar S, Kaushal JB (2024). Hydroxychloroquine interaction with phosphoinositide 3-kinase modulates prostate cancer growth in bone microenvironment: In vitro and molecular dynamics based approach. Int J Biol Macromol.

[159] Tewari D, Patni P, Bishayee A, Sah AN, Bishayee A (2022). Natural products targeting the PI3K-Akt-mTOR signaling pathway in cancer: A novel therapeutic strategy. Semin Cancer Biol.

[160] Cheng Y, Gao Y, Li J, Rui T, Li Q, Chen H (2023). TrkB agonist N-acetyl serotonin promotes functional recovery after traumatic brain injury by suppressing ferroptosis via the PI3K/Akt/Nrf2/Ferritin H pathway. Free Radic Biol Med.

[161] Liu Y, Chou F-J, Lang F, Zhang M, Song H, Zhang W (2023). Protein Kinase B (PKB/AKT) Protects IDH-Mutated Glioma from Ferroptosis via Nrf2. Clin Cancer Res.

[162] Shen M, Cao S, Long X, Xiao L, Yang L, Zhang P (2024). DNAJC12 causes breast cancer chemotherapy resistance by repressing doxorubicin-induced ferroptosis and apoptosis via activation of AKT. Redox Biol.

[163] Lan D, Yao C, Li X, Liu H, Wang D, Wang Y (2022). Tocopherol attenuates the oxidative stress of BMSCs by inhibiting ferroptosis through the PI3k/AKT/mTOR pathway. Front Bioeng Biotechnol.

[164] Lan D, Qi S, Yao C, Li X, Liu H, Wang D (2022). Quercetin protects rat BMSCs from oxidative stress via ferroptosis. J Mol Endocrinol.

[165] Huang Z-N, Wang Z-Y, Cheng X-F, Huang Z-Z, Han Y-L, Cui Y-Z (2023). Melatonin alleviates oxidative stress-induced injury to nucleus pulposus-derived mesenchymal stem cells through activating PI3K/Akt pathway. J Orthop Translat.

[166] Nan L-P, Wang F, Ran D, Zhou S-F, Liu Y, Zhang Z (2020). Naringin alleviates H2O2-induced apoptosis via the PI3K/Akt pathway in rat nucleus pulposus-derived mesenchymal stem cells. Connect Tissue Res.

[167] Li Y, Zhang K, Ai X, Zhang Q, Jiang L, Long J (2023). A Biomimetic Peptide Functions as Specific Extracellular Matrix for Quiescence of Stem Cells against Intervertebral Disc Degeneration. Small.

[168] Huang D, Peng Y, Ma K, Qing X, Deng X, Li Z (2020). Puerarin Relieved Compression-Induced Apoptosis and Mitochondrial Dysfunction in Human Nucleus Pulposus Mesenchymal Stem Cells via the PI3K/Akt Pathway. Stem Cells Int.

[169] Fejzo M, Rocha N, Cimino I, Lockhart SM, Petry CJ, Kay RG (2024). GDF15 linked to maternal risk of nausea and vomiting during pregnancy. Nature.

[170] Ferreira JP, Packer M, Butler J, Filippatos G, Pocock SJ, Januzzi JL (2024). Growth differentiation factor-15 and the effect of empagliflozin in heart failure: Findings from the EMPEROR program. Eur J Heart Fail.

[171] Takahashi N, Cho P, Selfors LM, Kuiken HJ, Kaul R, Fujiwara T (2020). 3D Culture Models with CRISPR Screens Reveal Hyperactive NRF2 as a Prerequisite for Spheroid Formation via Regulation of Proliferation and Ferroptosis. Mol Cell.

[172] Li W, Li W, Zhang W, Wang H, Yu L, Yang P (2023). Exogenous melatonin ameliorates steroid-induced osteonecrosis of the femoral head by modulating ferroptosis through GDF15-mediated signaling. Stem Cell Res Ther.

[173] Li X, Sun H, Zhang L, Liang H, Zhang B, Yang J (2024). GDF15 attenuates sepsis-induced myocardial dysfunction by inhibiting cardiomyocytes ferroptosis via the SOCS1/GPX4 signaling pathway. Eur J Pharmacol.

[174] Chen L, Qiao L, Bian Y, Sun X (2020). GDF15 knockdown promotes erastin-induced ferroptosis by decreasing SLC7A11 expression. Biochem Biophys Res Commun.

[175] Wu Z, Xi Q, Zhao Q, Zhu S (2024). GDF11 OVEREXPRESSION ALLEVIATES SEPSIS-INDUCED LUNG MICROVASCULAR ENDOTHELIAL BARRIER DAMAGE BY ACTIVATING SIRT1/NOX4 SIGNALING TO INHIBIT FERROPTOSIS. Shock.

[176] Zhu C, Zhou Q, Wang Z, Zhang J, Xu C, Ruan D (2023). Growth differentiation factor 5 inhibits lipopolysaccharide-mediated pyroptosis of nucleus pulposus mesenchymal stem cells via RhoA signaling pathway. Mol Biol Rep.

[177] Gadomski SJ, Mui BWH, Gorodetsky R, Paravastu SS, Featherall J, Li L (2024). Time- and cell-specific activation of BMP signaling restrains chondrocyte hypertrophy. iScience.

[178] Liu J, Pei C, Jia N, Han Y, Zhao S, Shen Z (2025). Preconditioning with Ginsenoside Rg3 mitigates cardiac injury induced by high-altitude hypobaric hypoxia exposure in mice by suppressing ferroptosis through inhibition of the RhoA/ROCK signaling pathway. J Ethnopharmacol.

[179] He Q, Zhou Y, Wu L, Huang L, Yuan Y, Flores JJ (2024). Inhibition of acid-sensing receptor GPR4 attenuates neuronal ferroptosis via RhoA/YAP signaling in a rat model of subarachnoid hemorrhage. Free Radic Biol Med.

[180] Ma T, Liu C, Zhao Q, Zhang Y, Xiao L (2024). Decellularized nucleus pulposus matrix/chitosan hybrid hydrogel combined with nucleus pulposus stem cells and GDF5-loaded microspheres for intervertebral disc degeneration prevention. Mol Med.

[181] Okoro PD, Frayssinet A, De Oliveira S, Rouquier L, Miklosic G, D'Este M (2023). Combining biomimetic collagen/hyaluronan hydrogels with discogenic growth factors promotes mesenchymal stroma cell differentiation into Nucleus Pulposus like cells. Biomater Sci.

[182] Brown CW, Amante JJ, Chhoy P, Elaimy AL, Liu H, Zhu LJ (2019). Prominin2 Drives Ferroptosis Resistance by Stimulating Iron Export. Dev Cell.

[183] Maggiorani D, Santin Y, Formoso K, Drapé E, Martini H, Brun S (2024). Identification of Prominin-2 as a new player of cardiomyocyte senescence in the aging heart. Aging Cell.

[184] Hu M, Yang J, Tan Z (2024). ATF1 promotes ferroptosis resistance in lung cancer through enhancing mRNA stability of PROM2. Exp Cell Res.

[185] An J, Shi J, Yang C, Luo J, Li Y, Ren J (2024). Regulation of tumorigenesis and ferroptosis in non-small cell lung cancer by a novel BBOX1-AS1/miR-326/PROM2 axis. Mol Cell Biochem.

[186] Paris J, Wilhelm C, Lebbé C, Elmallah M, Pamoukdjian F, Héraud A (2024). PROM2 overexpression induces metastatic potential through epithelial-to-mesenchymal transition and ferroptosis resistance in human cancers. Clin Transl Med.

[187] Adamiec-Organisciok M, Wegrzyn M, Cienciala L, Sojka D, Nackiewicz J, Skonieczna M (2023). Compensative Resistance to Erastin-Induced Ferroptosis in GPX4 Knock-Out Mutants in HCT116 Cell Lines. Pharmaceuticals (Basel).

[188] Belavgeni A, Bornstein SR, Linkermann A (2019). Prominin-2 Suppresses Ferroptosis Sensitivity. Dev Cell.

[189] Brown CW, Chhoy P, Mukhopadhyay D, Karner ER, Mercurio AM (2021). Targeting prominin2 transcription to overcome ferroptosis resistance in cancer. EMBO Mol Med.

[190] Cao S, Garcia SF, Shi H, James EI, Kito Y, Shi H (2024). Recognition of BACH1 quaternary structure degrons by two F-box proteins under oxidative stress. Cell.

[191] Amaral EP, Namasivayam S, Queiroz ATL, Fukutani E, Hilligan KL, Aberman K (2024). BACH1 promotes tissue necrosis and Mycobacterium tuberculosis susceptibility. Nat Microbiol.

[192] Kashif M, Yao H, Schmidt S, Chen X, Truong M, Tüksammel E (2023). ROS-lowering doses of vitamins C and A accelerate malignant melanoma metastasis. Redox Biol.

[193] Su Z, Kon N, Yi J, Zhao H, Zhang W, Tang Q (2023). Specific regulation of BACH1 by the hotspot mutant p53R175H reveals a distinct gain-of-function mechanism. Nat Cancer.

[194] Nishizawa H, Matsumoto M, Yamanaka M, Irikura R, Nakajima K, Tada K (2024). BACH1 inhibits senescence, obesity, and short lifespan by ferroptotic FGF21 secretion. Cell Rep.

[195] von Mässenhausen A, Schlecht MN, Beer K, Maremonti F, Tonnus W, Belavgeni A (2024). Treatment with siRNAs is commonly associated with GPX4 up-regulation and target knockdown-independent sensitization to ferroptosis. Sci Adv.

[196] Alvarado-Velez M, Enam SF, Mehta N, Lyon JG, LaPlaca MC, Bellamkonda RV (2021). Immuno-suppressive hydrogels enhance allogeneic MSC survival after transplantation in the injured brain. Biomaterials.

[197] Li M, Tian J, Yu K, Liu H, Yu X, Wang N (2024). A ROS-responsive hydrogel incorporated with dental follicle stem cell-derived small extracellular vesicles promotes dental pulp repair by ameliorating oxidative stress. Bioact Mater.

[198] Hu Y, Xing J, Zhang H, Pang X, Zhai Y, Cheng H (2024). Electroacoustic Responsive Cochlea-on-a-Chip. Adv Mater.

[199] He Z, Sun C, Ma Y, Chen X, Wang Y, Chen K (2024). Rejuvenating Aged Bone Repair through Multihierarchy Reactive Oxygen Species-Regulated Hydrogel. Adv Mater.

[200] Luo L, Li Y, Bao Z, Zhu D, Chen G, Li W (2024). Pericardial Delivery of SDF-1α Puerarin Hydrogel Promotes Heart Repair and Electrical Coupling. Adv Mater.

[201] Wang W, Liu Q, Yang Q, Fu S, Zheng D, Su Y (2024). 3D-printing hydrogel programmed released exosomes to restore aortic medial degeneration through inhibiting VSMC ferroptosis in aortic dissection. J Nanobiotechnology.

[202] Xu Y, Luo Y, Weng Z, Xu H, Zhang W, Li Q (2023). Microenvironment-Responsive Metal-Phenolic Nanozyme Release Platform with Antibacterial, ROS Scavenging, and Osteogenesis for Periodontitis. ACS Nano.

[203] Li Z, Zhao T, Ding J, Gu H, Wang Q, Wang Y (2023). A reactive oxygen species-responsive hydrogel encapsulated with bone marrow derived stem cells promotes repair and regeneration of spinal cord injury. Bioact Mater.

[204] Luo L, Gong J, Wang Z, Liu Y, Cao J, Qin J (2022). Injectable cartilage matrix hydrogel loaded with cartilage endplate stem cells engineered to release exosomes for non-invasive treatment of intervertebral disc degeneration. Bioact Mater.

[205] Dong Z, Liang P, Guan G, Yin B, Wang Y, Yue R (2022). Overcoming Hypoxia-Induced Ferroptosis Resistance via a 19 F/1 H-MRI Traceable Core-Shell Nanostructure. Angew Chem Int Ed Engl.

[206] Korupalli C, Kuo C-C, Getachew G, Dirersa WB, Wibrianto A, Rasal AS (2023). Multifunctional manganese oxide-based nanocomposite theranostic agent with glucose/light-responsive singlet oxygen generation and dual-modal imaging for cancer treatment. J Colloid Interface Sci.

[207] Wang X, Yu L, Duan J, Chang M, Hao M, Xiang Z (2024). Anti-Stress and Anti-ROS Effects of MnOx-Functionalized Thermosensitive Nanohydrogel Protect BMSCs for Intervertebral Disc Degeneration Repair. Adv Healthc Mater.

[208] Hoang DM, Pham PT, Bach TQ, Ngo ATL, Nguyen QT, Phan TTK (2022). Stem cell-based therapy for human diseases. Signal Transduct Target Ther.

[209] Zhou T, Yuan Z, Weng J, Pei D, Du X, He C (2021). Challenges and advances in clinical applications of mesenchymal stromal cells. J Hematol Oncol.

[210] Li J, Wu Z, Zhao L, Liu Y, Su Y, Gong X (2023). The heterogeneity of mesenchymal stem cells: an important issue to be addressed in cell therapy. Stem Cell Res Ther.

[211] Lee J, Yesilkanal AE, Wynne JP, Frankenberger C, Liu J, Yan J (2019). Effective breast cancer combination therapy targeting BACH1 and mitochondrial metabolism. Nature.

[212] Pan C, Cai Q, Li X, Li L, Yang L, Chen Y (2022). Enhancing the HSV-1-mediated antitumor immune response by suppressing Bach1. Cell Mol Immunol.

